# Mining the Metabolome and the Agricultural and Pharmaceutical Potential of Sea Foam-Derived Fungi

**DOI:** 10.3390/md18020128

**Published:** 2020-02-22

**Authors:** Ernest Oppong-Danquah, Cristina Passaretti, Orazio Chianese, Martina Blümel, Deniz Tasdemir

**Affiliations:** 1 GEOMAR Centre for Marine Biotechnology (GEOMAR-Biotech), Research Unit Marine Natural Products Chemistry, GEOMAR Helmholtz Centre for Ocean Research Kiel, Am Kiel-Kanal 44, 24106 Kiel, Germany; eoppong-danquah@geomar.de (E.O.-D.); cr.passaretti92@gmail.com (C.P.); ory_oi91@hotmail.it (O.C.); mbluemel@geomar.de (M.B.); 2 Faculty of Mathematics and Natural Science, Kiel University, Christian-Albrechts-Platz 4, 24118 Kiel, Germany

**Keywords:** sea foam, marine fungi, metabolomics, molecular network, phytopathogen, antimicrobial activity

## Abstract

Sea foam harbors a diverse range of fungal spores with biological and ecological relevance in marine environments. Fungi are known as the producers of secondary metabolites that are used in health and agricultural sectors, however the potentials of sea foam-derived fungi have remained unexplored. In this study, organic extracts of six foam-derived fungal isolates belonging to the genera *Penicillium*, *Cladosporium*, *Emericellopsis* and *Plectosphaerella* were investigated for their antimicrobial activity against plant and human pathogens and anticancer activity. In parallel, an untargeted metabolomics study using UPLC-QToF–MS/MS-based molecular networking (MN) was performed to unlock their chemical inventory. *Penicillium* strains were identified as the most prolific producers of compounds with an average of 165 parent ions per strain. In total, 49 known mycotoxins and functional metabolites were annotated to specific and ubiquitous parent ions, revealing considerable chemical diversity. This allowed the identification of putative new derivatives, such as a new analog of the antimicrobial tetrapeptide, fungisporin. Regarding bioactivity, the *Penicillium* sp. isolate 31.68F1B showed a strong and broad-spectrum activity against seven plant and human pathogens, with the phytopathogen *Magnaporthe oryzae* and the human pathogen *Candida albicans* being the most susceptible (IC_50_ values 2.2 and 6.3 µg/mL, respectively). This is the first study mining the metabolome of the sea foam-derived fungi by MS/MS-based molecular networking, and assessing their biological activities against phytopathogens.

## 1. Introduction

Sea foam has been known as a rich source of fungi over several decades [1]. Foams commonly appear as discolored patches on the water surface, and were long believed to be a consequence of pollution from human activities [2]. However, the presence of foam on nutrient-rich waters from rainforests and other pristine marine environments confirmed that it can also occur naturally [3]. Sea foam is formed by the churning effect of waves on seawater rich in dissolved organic matter (such as proteins, lipids and lignins), which acts as surface-active agents or surfactants. As the waves break upon the shoreline, the surface-active agents trap air in lamellae that aggregates to form the sea foam [4]. Naturally-occurring sea foam may contain humic substances from the offshore breakdown of algal bloom [5]. 

In contrast to natural sea foam, anthropogenic impacts associated with industrial and farming activities result in marked levels of phosphates from fertilizers, as well as organic and inorganic detergents in the seawater, contributing to sea foam formation [6,7]. Hence, sea foam has no stable composition, but represents a highly variable and dynamic environment. Sea foams are reported to play important roles in the transfer of surface-active pollutants and toxins, which imposes high stress levels on the organisms thriving in them [8]. Simultaneously, the high organic matter content renders sea foam an interesting ecological niche for heterotrophic organisms, such as fungi [3]. Sea foam has recorded very high levels of organic carbon, originating from amino acids, phenols, aldoses and deoxy sugars [3]. As an enriched source of organic carbons, sea foam is an excellent accumulator of fungal strains.

Sea foam does not only comprise marine, but also terrestrial fungi, as propagules of terrestrial fungi are transported off the shores by tidal waves or wind, and then trapped in foams [9,10]. Due to these varying sources of fungi propagules, combined with the high stress level due to the highly dynamic nature of their environment, sea foam-derived fungi could serve as a resource for novel functional metabolites with unique chemical and biological properties. However, sea foam has been sparsely studied, and the majority of investigations have focused only on the microbial diversity and the multifunctional components involved in its physical and biochemical stability [9,11]. To our knowledge, the only study carried out on the chemical constituents of the sea foam-derived fungi was conducted by Overy et al. (2014) on foam material collected around the pristine coast of Prince Edward Island (Canada) [4]. This study led to the isolation of 48 fungal species belonging to unknown taxa, ubiquitous strains, as well as isolates historically associated with terrestrial substrata (e.g. plants and soil), including *Alternaria* sp., *Cladosporium* sp., *Penicillium* sp., *Epicoccum* sp. and *Aspergillus* sp. The authors further reported natural products that inhibited nosocomial pathogens and cancer cell lines [4], pointing out the untapped potential of sea foam-derived fungi in marine biodiscovery. 

As an understudied and stressful niche, sea foam has the potential to represent a unique source of fungi for novel secondary metabolites with a fascinating bioactivity profile. Hence, the current study aimed at investigating the metabolome of the sea foam-derived fungi. An untargeted metabolomics study using UPLC-QToF–MS/MS based molecular networking (MN) was performed on the EtOAc extracts of six fungal strains isolated from the sea foam sampled [12] from the Windebyer Noor (Baltic Sea), Germany. This resulted in the annotation of diverse compounds, including meroterpenoids, polyketides, peptides, alkaloids and many putative new compounds. The extracts were tested for antimicrobial activity against a panel of plant pathogens, human pathogens (including the bacterial ESKAPE panel and fungi), and for their anticancer activity.

## 2. Results

### 2.1. Selection of Isolates and Phylogenetic Analysis

In our previous study, we reported the isolation of fungi from seawater, sea foam and sediment from the Windebyer Noor (Baltic Sea) [12]. From the total of sixteen isolated and identified sea foam-derived fungi [12], six representative strains belonging to the genera *Penicillium* (three strains), *Cladosporium* (one strain), *Plectosphaerella* (one strain) and *Emericellopsis* (one strain), were investigated for their chemical diversity and biological activity. These six isolates were selected according to their phylogenetic classification (Figures S1 and S2). Herein we performed a new BLAST search with the sequences obtained in a previous study [12]. These sequences were submitted to BLAST twice: first, searching for highly similar sequences, and second, limiting the search to type strains (Table S1). Sequences from the five closest relatives from both searches were used to construct the phylogenetic trees (Figure S1 (ITS) and Figure S2 (18S rRNA gene)). For one isolate (*Penicillium* sp. 36.97 F1C), repeated PCRs did not yield an ITS fragment; therefore, we amplified and sequenced the 18S rRNA gene (Figure S2), which identified the isolate as a *Penicillium* sp. Table 1 shows the selected fungi and their GenBank accession numbers.

### 2.2. Metabolomics

**Figure 1:**
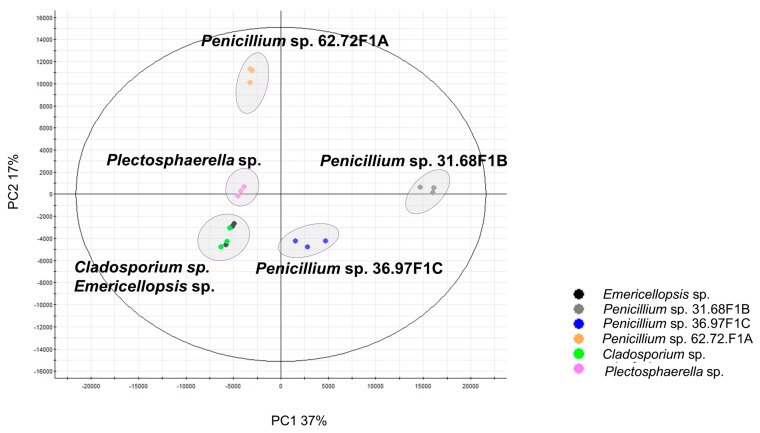
Principal component analysis (PCA) scores plot of all six fungal extracts showing discrimination in chemical diversity.

For conducting metabolomics studies, the selected fungi were cultivated on potato dextrose agar medium (PDA). PDA was the culture medium of choice, based on a pilot experiment performed in previous study [12]. Eleven different cultivation media were investigated to support the growth of fungi and their production of diverse metabolites in mono- and co-cultures, PDA emerging as the best one [12]. Cultures of the sea foam-derived fungi grown on solid PDA medium were extracted with EtOAc and analyzed by high resolution tandem mass spectrometry (UPLC-HRMS/MS). Triplicate profiles were combined for each strain for comparative multivariate analysis and metabolomics, including molecular networks (MNs). The principal component analysis (PCA) scores plot of the HRESIMS data (Figure 1) showed a variation in the chemical profiles of the selected fungi. *Cladosporium* sp. and *Emericellopsis* sp. clustered closely together, while *Plectosphaerella* sp. clustered separately. Notably, the three *Penicillium* strains showed differential chemical profiles by clustering separately (Figure 1). On the contrary, phylogenetic analyses (Figures S1 and S2) revealed that these strains were closely related, prompting a separate analysis to investigate their chemical diversity. 

**Figure 2:**
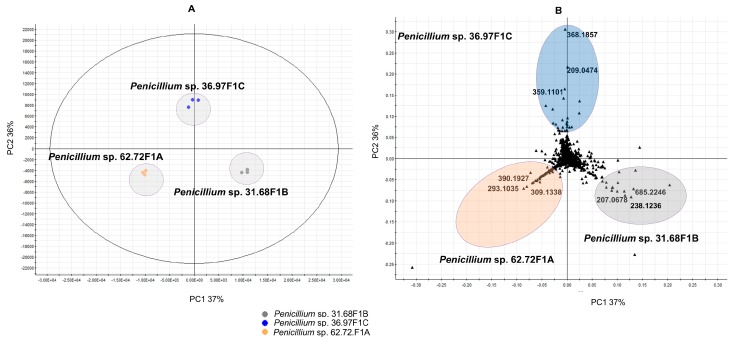
(**A**) PCA scores plot of the three *Penicillium* strains (**B**) PCA loadings plot showing regions (blue: isolate 36.97F1C; orange: isolate 62.72F1A and grey: isolate 31.68F1B) of highly specific features among the *Penicillium* strains. Three metabolites each, contributing to the discrimination (displayed *m/z* values) of the three *Penicillium* strains, are highlighted in the PCA loadings plot and listed in Table 2 as examples. All other discriminatory metabolites annotated are shown in Table S2.

To investigate the intra-*Penicillium* genus chemical diversity, the three *Penicillium* strains were analyzed separately in a PCA scores plot (Figure 2A). The scores PC1 37% and PC2 36% clearly revealed dissimilarities in the metabolome of the three *Penicillii*.

A PCA loadings plot (Figure 2B), which shows the distribution of the metabolites (*m/z*) for the respective *Penicillium* spp., was also generated. The differently-colored areas in the loadings plot geometrically correlate with the respective areas in the PCA scores plot (Figure 2A). Features representing metabolites (*m/z*) contributing to the discrimination of the three *Penicillium* strains (exemplified with three peak ions each) are indicated in the loadings plot (Figure 2B), and also their putative annotations are listed in Table 2. All other discriminatory metabolites annotated in this study can be found in Table S2. 

Dereplication of the discriminatory metabolites identified from the loadings plot for the isolate 31.68F1B identified the ion *m/z* [M + H]^+^ 207.0678 as the benzopyrane derivative, 7-hydroxy-2-(hydroxymethyl)-5-methyl-4H-1-benzopyran-4-one. The ion *m/z* [M + H]^+^ 238.1236 could not be identified, because the search returned no hit of microbial origin. As shown in Table 2, other annotated discriminatory metabolites include the tropolone viticolin C, indole alkaloid brevianamide E, hydroxyl derivative of mycophenolic acid for *Penicillium* sp. isolate 36.97F1C, and the polyketide italicic acid and diketopiperazine tryhistatin for *Penicillium* sp. isolate 62.72F1A. 

Supplementary Table S2 details all metabolites putatively annotated in this study. To facilitate the dereplication of all discriminatory and ubiquitous metabolites, we employed the MS^2^-based molecular networking (MN) tool. 

#### 2.2.1. Molecular Network Analyses of the *Penicillium* Strains

**Figure 3:**
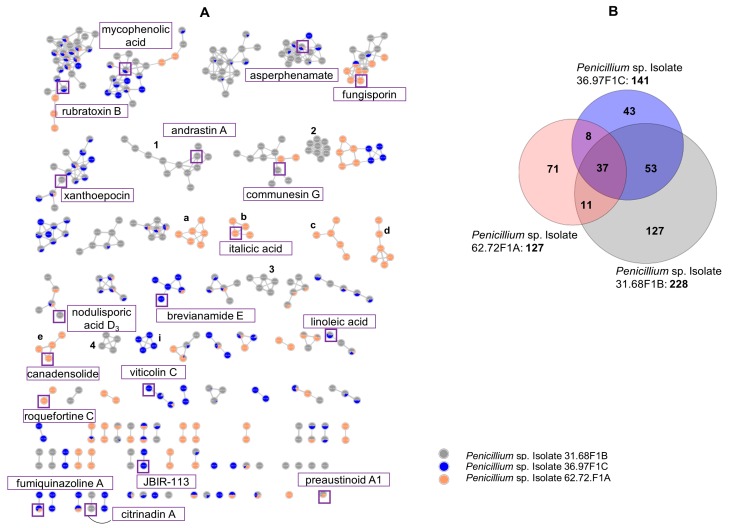
(**A**) Molecular network (MN) of extracts from three *Penicillium* strains. Annotated compounds are displayed in Table S2. Nodes represent parent ions detected in the crude extracts of the fungi. Nodes that are highlighted in boxes, also named in the rectangles close to them, indicate representatives of that compound molecular family (**B**) Venn diagram displaying specific and shared parent ions detected in the culture extracts of three *Penicillium* strains.

MN was generated for three *Penicillium* spp. to facilitate the dereplication of metabolites, and also to serve as a comparative tool to investigate their chemical diversity. Analysis of the global MN for all three *Penicillium* strains revealed 350 nodes (Figure 3A), representing parent ions originating from the crude extracts. Nodes from the blank media extraction were removed. In addition to the automatic comparison of the dataset against the publicly-available spectral databases on the Global Natural Product Social Molecular Network (GNPS) platform, we conducted manual dereplication. A total of 18 annotated compound classes included some known mycotoxins of *Penicillium* spp., such as the meroterpenoid mycophenolic acid [13] and the cyclic tetrapeptide fungisporin [14] (Figure 3A). Others included the meroterpenoids andrastin A [15] and prestaunoid A1 [16], polyketides italicic acid [17], xanthoepocin [18], rubratoxin B [19] and canadensolide [20], indole alkaloids brevianamide A [21], communesin G [22] and fumiquinazoline A [23], diketopiperazine roquefortine C [24], phenylalanine derivative asperphenamate [25], tropolone viticolin C [26], pentacyclic alkaloid citrinadin A [27] and the depsipeptide JBIR-113 [28] types of compounds.

MN also revealed interesting patterns for the comparative analysis of the three *Penicillium* strains. Thirty-six molecular families (MFs, cluster of more than two nodes) distributed among the strains were identified. Four MFs (‘1–4’ in Figure 3A) were specific to *Penicillium* sp. isolate 31.68F1B. These included the andrastin A and three unidentified clusters (2–4). *Penicillium* sp. isolate 62.72F1A displayed five strain-specific MFs (‘a–e’ in Figure 3A), which included the polyketides italicic acid and canadensolide classes of compounds. *Penicillium* sp. isolate 36.97F1C showed the least number of strain-specific MFs displaying only one (‘i’ in Figure 3A), which could not be annotated. Many MFs were shared by all three strains, such as the asperphenamate and mycophenolic acid clusters. Some strain-specific parent ions were annotated in shared clusters. For example, the indole alkaloid communesin G and polyketide xanthoepocin were specific to *Penicillium* sp. isolate 31.68F1B, and the cyclic tetrapeptide fungisporin was specific to *Penicillium* sp. isolate 62.72F1A. A conjugated linoleic acid cluster representing fatty acid type primary metabolites was shared by all three *Penicillium* strains.

A Venn diagram based on the MN showed the parent ion distribution among the three *Penicillium* strains (Figure 3B). The highest number of nodes was observed in *Penicillium* sp. isolate 31.68F1B (228), followed by *Penicillium* isolate 36.97F1C (141 nodes). *Penicillium* isolate 62.72F1A showed the least number of nodes (127). In total, 241 parent ions (69%) showed a strain-specific distribution (isolate 31.68F1B: 127, isolate 36.97F1C: 43 and isolate 62.72F1A: 71 parent ions, respectively). This was further indicative of the unique chemistry produced by different *Penicillium* strains. *Penicillium* sp. isolate 36.97F1C showed a similarity of 97.77% to both type species *P. commune* CBS 343.51 T and *P. limosum* CBS 339.97 T (Table S1). The other two strains, *Penicillium* spp. 31.68F1B and 62.72F1A both showed 100% similarity to the type strain *P. bialowiezense* CBS 227.28T (Table S1). Nonetheless, 109 nodes were shared by at least two strains, and 37 nodes (10.5%) shared by all three strains. 

Dereplication of the parent ions was more efficient using MN, which enables easy annotation of the derivatives of known compounds. As an example, a MF that was shared by all three *Penicillium* strains was the meroterpenoid mycophenolic acid A cluster. The antibiotic mycophenolic acid (present in all *Penicillii*) was identified based on the high-resolution mass spectrometry (HRMS) predicted formula for *m/z* [M + H]^+^ 321.1342 as C_17_H_21_O_6_ (1.2 ppm error), and its MS/MS spectrum. The MS/MS spectrum was compared with the library spectrum of mycophenolic acid (Figure S3), resulting in a perfect match. The large number of nodes clustering with mycophenolic acid (annotated parent ion, Figure 3A) was indicative of several additional analogs and derivatives. 

The cluster analysis (Figure 3A) based on the differences in atomic mass units (amus) relative to the annotated mycophenolic acid node revealed the presence of a methylated analog (*m/z* 335.1340 in isolates 31.68F1B and 36.97F1C), which was annotated as mycophenolic acid methyl ester [13] (Table S2). Other nodes in this cluster included the demethylated (*m/z* 307.1001 specific to isolate 31.68F1B), dehydrogenated (*m/z* 319.1010 in isolates 31.68F1B and 36.97F1C) and dehydroxylated (*m/z* 303.1070 in all *Penicillii*) derivatives of mycophenolic acid, as well as a loss of 26 amu derivative (*m/z* 295.1010, corresponding to loss of an ethylene group specific to isolate 31.68F1B). Some of these analogs are putatively new natural products.

Another MF shared by all three *Penicillium* strains was the fungisporin cluster (Figure 3A). The cyclic tetrapeptide fungisporin (only identified in *Penicillium* isolate 62.72 F1A) was also annotated based on the molecular formula prediction from HRMS *m/z* [M + H]^+^ 493.2815 (C_28_H_37_N_4_O_4_, 0.6 ppm error) and MS/MS similarity to that published in the literature [14]. Cyclic peptides have characteristically unexplained spectral intensities observed in their MS/MS spectra. Cyclic peptides undergo collision-induced dissociation to produce ‘nondirect sequence ions’, (NDS) due to the scrambling of sequence information, as opposed to typical fragmentation termed as a ‘direct sequence ion’ (DS) [29]. For small cyclic peptides, such as tetrapeptides, we employed a simple approach to determine the amino acid sequence. According to the conventional pathway for fragmentation, cyclic peptide rings are randomly opened at each amide bond, yielding different linear peptides, which are further fragmented to yield NDS and DS ions [30]. 

**Figure 4:**
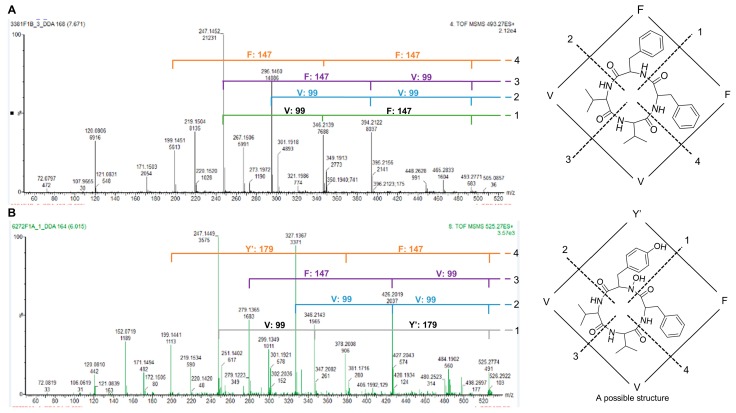
Cyclic tetrapeptides undergo random ring opening at each amide bond to yield linear peptides which fragment to yield nondirect sequence (NDS) ions and direct sequence (DS) ions. The first two fragments from each ring opening point (1, 2, 3 and 4) are annotated, and the sequence of the cyclo-tetrapeptide is consequently predicted. (**A**) MS/MS spectrum of node *m/z* 493.2730 annotated as fungisporin. (**B**) Annotated MS/MS spectrum of the putative new analog of fungisporin *m/z* 525.2654. V, valine; F, phenylalanine; Y, tyrosine; Y’, putatively identified as N-hydroxyl-tyrosine (as shown in Figure 5B) or β-hydroxyl-tyrosine.

From our MS/MS experiments, we could identify two DS ions for every possible break point in the cyclic tetrapeptides (Figure 4). Based on the sequences of two defined amino acid fragments, a logical sequence for the cyclic peptide was proposed, which confirmed the annotation fungisporin as cyclo–FFVV peptide (Figure 4) [14]. Additional analogs analyzed in this cluster included the known compounds *m/z* 509.2691 as cyclo–YFVV (common to isolates 62.72F1A and 36.97F1C) [14] and *m/z* 532.2857 as cyclo–FWVV (common to isolates 62.72F1A and 31.68F1B) [14]. The fungisporin analog *m/z* 525.2654 (specific to isolate 62.72F1A) was identified as a putatively new cyclic tetrapeptide with a cyclo -Y’FVV, where Y’ is a modified amino acid with a monoisotopic residue mass of 179. It has a similar sequence with node 509.2691, but the tyrosine (Y) is replaced with a hydroxyl derivative (Y’) (Figure 4).

Of the nine strain-specific MFs (‘a–e’, ‘1–4’ and ‘i’, Figure 3A), the andrastin A cluster was the biggest, comprising 12 parent ions originating from the *Penicillium* sp. isolate 31.68F1B. Meroterpenoid andrastin A (*m/z* [M + H]^+^ 487.2612) was annotated as a result of database comparison against the GNPS spectral library. Other nodes in this MF putatively annotated included andrastin B (*m/z* [M + H]^+^ 489.2671) and andrastin D (*m/z* [M + H]^+^ 429.2430). The remaining nodes in the meroterpenoid MF were not assigned to known natural products, and remain potentially new.

#### 2.2.2. Molecular Network Analyses of the *Cladosporium* and *Emericellopsis* sp. 

**Figure 5:**
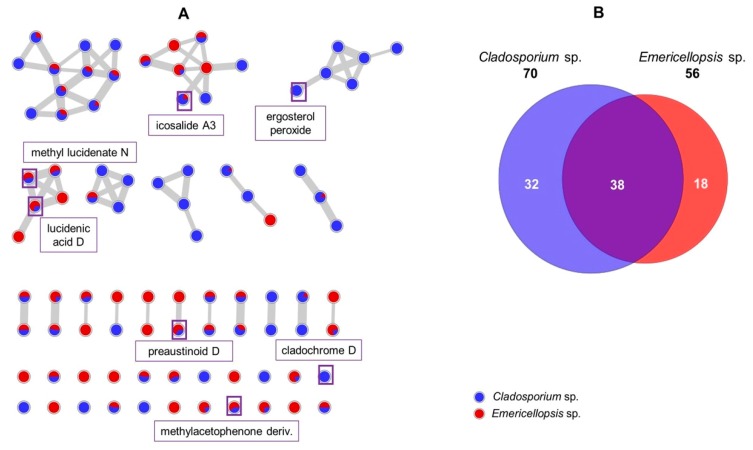
(**A**) MN of *Cladosporium* sp. and *Emericellopsis* sp. highlighting annotated parent ions. Nodes highlighted in boxes, also named in the rectangles close to them, indicate representatives of that compound molecular family (**B**) Euler diagram generated from the MN showing strain specific ions and shared ions between the two strains.

Although two fungal isolates emerged in two different clades in the phylogenetic analysis (Figure S1) belonging to the orders Capnodiales (*Cladosporium* sp.) and Hypocreales (*Emericellopsis* sp.), the PCA analysis (Figure 1) revealed a high similarity in their chemical diversity. As a result, the metabolomes of *Cladosporium* sp. and *Emericellopsis* sp. were also analyzed comparatively by MN (Figure 5) generated from the MS/MS experiments. Their UPLC chromatograms displayed poor chemical diversity with few distinct peaks (Figure S4). 

We putatively annotated the steroid ergosterol peroxide [31], perylenequinone cladochrome D [32], polyketide dihydroxy methylacetophenone [33], meroterpenoid preaustinoid D [34], triterpenoid lucidenic acid D [35] and cyclic peptide icosalide [26] classes of compounds. In total, 88 parent ions were observed in the MN. As visualized in the Euler diagram (Figure 5B), *Cladosporium* sp. contained more parent ions (70) than *Emericellopsis* sp. (56). As predicted from the PCA plot (Figure 1), more than a third (38 ions, equivalent to 43%) were shared parent ions. However, strain-specific ions were also observed, which were 32 ions for *Cladosporium* sp., and only 18 ions for *Emericellopsis* sp. These resulted in two MFs (more than two nodes in a cluster) specific to the *Cladosporium* sp. One MF was annotated as an ergosterol peroxide class of compounds, while the other was not annotated, and may represent putative new metabolites. *Emericellopsis* sp. showed no strain-specific MF, but more singletons (nodes without edges). Although the two taxonomically non-related fungal strains were surprisingly chemically related when cultured on PDA medium, *Cladosporium* sp. showed much higher chemical diversity than *Emericellopsis* sp. The largest MF, consisting of 11 parent ions, was shared by both strains. They were observed in the mass range of *m/z* 472 to 512 amu within t*_R_* 7–10 min, and with low intensities. This MF was however not annotated, and could represent putatively new metabolites.

#### 2.2.3. Molecular Network Analysis of *Plectosphaerella* sp.

**Figure 6:**
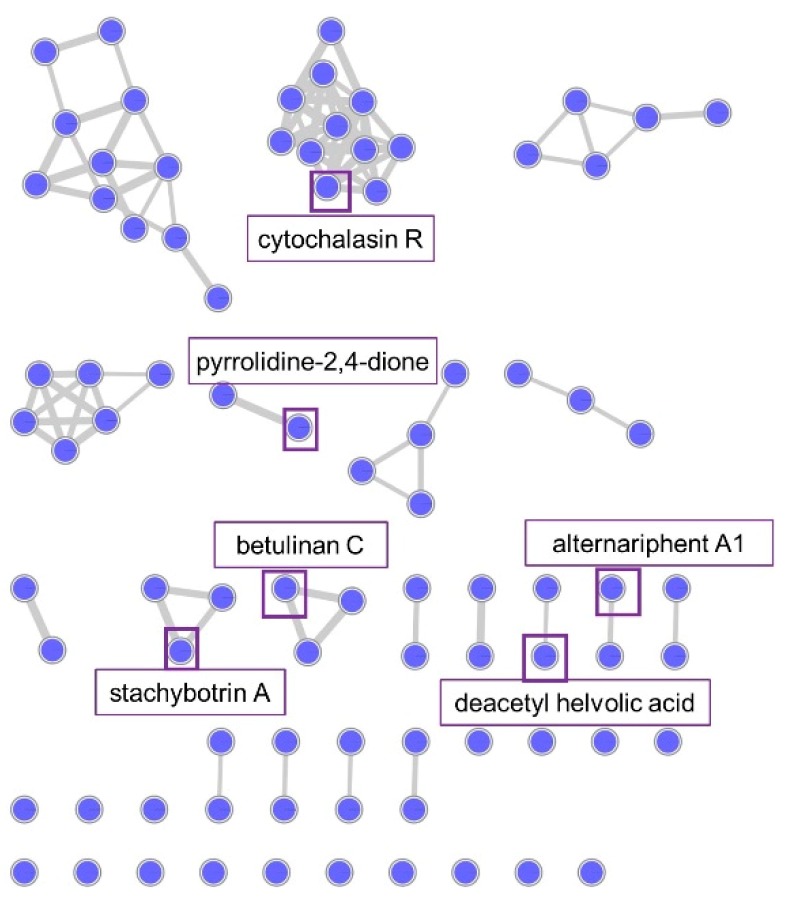
MN of *Plectosphaerella* sp. highlighting annotated parent ions. Nodes highlighted in boxes, also named in the rectangles close to them, indicate representatives of that compound MF.

The chemical diversity in the organic extract of *Plectosphaerella* sp. was also investigated using MN. After removing nodes associated with the culture medium and the solvent, the MN contained 85 parent ions (nodes) and 94 edges (chemical relatedness). These clustered into 8 MFs. About 20% (17 nodes) showed no association with edges, representing compounds with unique chemistry. Comparison against the GNPS database yielded no results, hence an extensive manual dereplication approach was adopted. This resulted in the annotation of about 10% of the parent ions (Figure 6). Some annotated ions included the indole alkaloid cytochalasin R [36], pyranone betulinan C [37], alkaloids, 3-acetyl-5-isopropyl-pyrrolidine-2,4-dione [38] and stachybotrin A [39], phenol derivative alternariphent A1 [40,41] and nortriterpenoid deacetyl helvolic acid [42]. 

### 2.3. Antimicrobial and Cytotoxic Activity

The crude extracts of the six selected strains obtained from the sea foam were tested against a panel of phytopathogens comprising four bacteria; *Pseudomonas syringae*, *Xanthomonas campestris*, *Erwinia amylovora* and *Ralstonia solanacearum*; two fungi; *Magnaporthe oryzae*, and *Botrytis cinerea*, and an oomycete *Phytophthora infestans*. The crude extract of *Penicillium* sp. 31.68F1B exhibited potent activity against the fungal plant pathogens *M. oryzae, P. infestans* and *B. cinerea* with IC_50_ values of 2.2, 4.3 and 13.8 µg/mL, respectively. *Penicillium* sp. isolate 36.97F1C exerted moderate activity against phytopathogens *P. infestans* and *M. oryzae*, with IC_50_ values of 12.9 and 31.6 µg/mL, respectively (Table 3). 

Organic fungal extracts were also evaluated for their inhibitory activity against eight human bacterial and fungal pathogens. The test systems comprised the ESKAPE panel pathogens, including drug-resistant bacteria ***E****nterococcus faecium,* methicillin–resistant ***S**taphylococcus aureus (MRSA), **K**lebsiella pneumoniae, **A**cinetobacter baumannii, **P**seudomonas aeruginosa, **E**scherichia coli*, and the fungi *Candida albicans* and *Cryptococcus neoformans*. The extracts that were tested at an initial concentration of 100 µg/mL exhibited varying degrees of inhibitory activities (Table S3). The IC_50_ values were determined for those extracts showing growth inhibitory activities of more than 70% (Table 3). *Penicillium* isolates 31.68F1B and 36.97F1C showed the broadest and strongest activities against the human Gram-positive bacterial and fungal pathogens.

Isolates 31.68F1B strongly inhibited *E. faecium*, MRSA (IC_50_ values of 15 and 31 µg/mL, respectively) and fungal pathogens *C. albicans* and *C. neoformans* (IC_50_ values of 6.3 and 9.2 µg/mL, respectively). Isolate 36.97F1C exerted moderate inhibition against the bacterium *E. faecium* (IC_50_ value 37.8 µg/mL) and the fungus *C. neoformans* (IC_50_ value 26.9 µg/mL). 

The extracts were also assessed for their inhibitory potential against the lung carcinoma cancer cell line A-549, the breast cancer cell line MB-231, and for general toxicity towards the human keratinocyte cell line HaCaT. There was no anticancer activity at the 100 µg/mL concentration, but also no toxicity towards the non-cancerous cell line, HaCaT. 

## 3. Discussion

We established a taxonomically representative collection of six out of 16 sea foam-derived fungi, to investigate their potential to produce bioactive secondary metabolites, and indexed the chemical inventory of the extracts by MN-aided dereplication. The sea foam-derived strains were cultured on potato dextrose agar (PDA), based on an initial screening for optimal cultivation media [12]. The fungi isolated in our previous study [12] were dominated by Ascomycetes, affiliated to the cosmopolitan strains of the *Penicillium, Cladosporium, Plectosphaerella* and *Emericellopsis* genera. With the exception of *Plectosphaerella,* all the other genera were also identified in a similar study, which showed the diversity in sea foam-derived fungi on the coast of the Canadian Prince Edward Island [4]. The literature shows that these species are common across a wide array of niches in both terrestrial and marine environments [43,44,45,46]. Similar to previous studies, the majority of sea foam fungal isolates found in this study are ubiquitous saprophytes [1,4]. They are likely to result from entrapped spores blown from neighboring terrestrial fields [47]. The sea foam-derived strains used in this study may therefore likely not to be ‘truly marine’. This is important in the metabolite dereplication process, as database searches covering both marine and terrestrial fungal compounds needed to be adopted. Crude extracts of the sea foam-derived fungi were chemically profiled by UPLC-ESIMS/MS. Spectral data were analyzed using multivariate analytical tools and MN. The PCA plot of the HRMS/MS data of all crude extracts revealed clear discrimination of all different strains, except *Cladosporium* sp. and *Emericellopsis* sp. The PCA scores plot also highlighted the reproducibility (in replicates) of the metabolites biosynthesized by the strains on the solid cultivation medium [48]. The strain discrimination observed in the PCA was indicative of the variability in secondary metabolite production. This is in agreement to the concept of microbial chemotaxonomy, which addresses the chemical variation among microbes [49]. Although it is largely debated among natural product scientists, microbial chemotaxonomy has been successfully implemented in some genera, such as *Penicillium*, *Alternaria* and *Hypoxylon* [50]. This result supports microbial chemotaxonomy.

MS/MS-based MN is an approach that has revolutionized the dereplication of complex natural product mixtures. It enables the visualization of large data sets as related compound clusters using algorithms to compare similarity in their fragmentation spectra [51]. MN is therefore an important complementary tool for comparative metabolomics. It was heavily employed in this study as a dereplication approach, and for comparing the chemical repertoire of strains within a genus. In parallel, the crude extracts of all selected fungi were screened against a panel of economically relevant plant pathogens, human pathogens and cancer cell lines for the evaluation of their bioactivity.

The genus *Penicillium* showed very diverse classes of compounds with about a 5% annotation rate compared to the 1.8% average annotation rate in untargeted metabolomics efforts [52]. This highlights the efficiency of MN as a dereplication tool. Furthermore, the clustering of the metabolites from the same molecular families in the networks offered in-depth information on the real chemical diversity and inventory of the fungi. Among the ubiquitous parent ions was the annotated phenylalanine derivative asperphenamate. Asperphenamate and its derivatives have been reported from the fungal genera *Penicillium* and *Aspergillus* with antimicrobial and anticancer activities [53,54]. These are rare linear amino acid esters derived from nonribosomal peptides [55]. Another parent ion identified in all three *Penicillium* strains was meroterpenoid mycophenolic acid, a polyketide-terpene hybrid composed of a phthalide moiety and a terpenoid side chain. 

With many reported biological activities (antiviral, antibacterial, antifungal and antitumor) of mycophenolic acid, it is a commercially-available immunosuppressant as a prodrug in the mofetil ester form [56]. These ubiquitous, annotated, bioactive metabolites may be responsible for the observed antimicrobial activities among the *Penicillium* strains (Table S3). In all, 31% (109 parent ions) were shared by a minimum of two *Penicillium* strains. However, not only the presence, but also the concentration, of bioactive metabolites in crude extracts, is critical for bioactivity screening. A study to quantitatively assess the secondary metabolites from grape berry-derived *Penicillium* strains revealed the differential production of the mycotoxin chaetoglobosin A in two species of *P. expansum* [57]. While *P. expansum* MK9/2009 produced 340 µg/L, *P. expansum* VZ19/2008 produced 3,455 µg/L [57] in the same cultivation medium and conditions. Different titers of bioactive metabolites in this study may also be responsible for the varying potency of the *Penicillium* strains with similar bioactive parent ions. 

The cyclic tetrapeptide fungisporin MF was also shared by all three *Penicillii*. The analysis of this cluster revealed 14 parent ions annotated to known and unknown compounds. One putatively new structure was proposed as an analog of the known compound cyclo – YFVV [14], in which the tyrosine is hydroxylated. Purification and analysis of this compound using NMR spectroscopy will be needed to confirm the structure. *Penicillium* sp. 31.68F1B exhibited the highest and broadest activities against the tested plant and human pathogens, and this correlates well with the chemical diversity visualized in the MN. *Penicillium* sp. isolate 31.68F1B showed four specific MFs (1–4 in Figure 3), and could be responsible for the broad activities observed for this strain. MF-1 was putatively annotated as meroterpenoid andrastin cluster (Figure 3A). Andrastins are RAS protein farnesyltransferase (relays proliferative signals) inhibitors, making them good anticancer candidates [15]. In a recent report comparing the metabolomics and transcriptomics of both solid-state and submerged cultivation of *Penicillium expansum*, the meroterpenoid andrastins and their gene transcripts were exclusively detected in the solid-state cultivation conditions [58]. This supports our annotation of andrastin, as the strains were grown on solid agar. Three other MFs (2–4 in Figure 3A) specific to *Penicillium* sp. isolate 31.68F1B, were not annotated, and represent putatively new classes of compounds. In addition, the exclusive presence of the indole alkaloid communesin G, with reported antibacterial and antifungal activity [22] and antifungal polyketide xanthoepocin [18], may also contribute to the potent antimicrobial activities observed in *Penicillium* sp. isolate 31.68F1B.

*Cladosporium* sp. and *Emericellopsis* sp. generally showed a lesser number of parent ions (Figure S4). *Cladosporium* sp. however contained more diverse compounds than *Emericellopsis* sp. The majority of parent ions (43%) were produced across species boundaries. One significant annotated ion is the perylenequinone pigment cladochrome E [32]. This class of compounds has attracted much attention because of their photodynamic and phytotoxic potentials [32]. The strain-specific cluster ergosterol peroxide is one of two clusters identified only in *Cladosporium* sp. This class of compounds have previously been reported from fungi and plants with anticancer, antibacterial and immunosuppressive activities [59]. The glycosylated form of the ergosterol peroxide was annotated as 5α,8α-epidioxy-24(R)-methylcholesta-6,22-dien-3β-D-glucopyranoside [31]. The bioactive polyketide dihydroxymethylacetophenone was annotated to another singleton which was shared by both *Cladosporium* and *Emericellopsis* strains [33,60]. The low activity against the microbial pathogens may stem from the low concentration of the putative bioactive compounds in the crude extract. It is however worth noting that the *Emericellopsis* sp. 25.88F1C showed only 95% ITS sequence similarity to its closest relative *Emericellopsis* sp. HQ649988.1, and hence may be a new species; further investigations will follow. Other annotated parent ions are detailed in Table S2. 

*Plectosphaerella* genus is not well studied for its chemical constituents. A literature search through the Dictionary of Natural Products (DNP) database [26] on the genus *Plectosphaerella* revealed only four compounds, which included the indole alkaloids, plectosphaeroic acid type of compounds and the trisulfide analog of the epipolythiodioxopiperazine gliocladine. Unfortunately, these reported compounds were not annotated in our study, probably due to various reasons, such as species differences, geographical and ecological differences, media variation and responsible gene cluster activation [61,62]. 

Our dereplication efforts however revealed the presence of the indole alkaloid cytochalasin R in *Plectosphaerella* sp. More than 100 cytochalasins, typified by an isoindole ring fused to a macrocyclic ring, have been isolated from several fungal sources [63]. Their reported activities include antimicrobial, anticancer, phytotoxic and antiparasitic [64]. Amidst the many compounds already reported in this class, some nodes in the cluster were not annotated, and may represent potentially novel compounds. Some other annotated parent ions include the phenolic compound alternariphent A1 [41], alkaloid stachybotrin A [39] and nortriterpenoid helvolic acid methyl ester [65] with reported antibacterial and antifungal activities (DNP). The minimal activities observed against the tested pathogens (Table S3) may be attributed to a low concentration of the bioactive compounds. Many parent ions from the *Plectosphaerella* sp. extract could not be annotated, and may also represent new natural products.

Sea foam is an ideal source of fungi, due to its high carbon and nutrient content. The present study indicates that sea foam-derived fungi are prolific producers of bioactive and chemically diverse metabolites from different classes, such as meroterpenes, alkaloids and peptides. Notably, this is only the second chemical study performed on sea foam-derived fungi, but remains as the first study applying a metabolomics approach by MN to investigate the comprehensive and comparative chemical composition of the sea foam-associated fungi. The combination of the MN using the GNPS platform and manual dereplication efforts significantly facilitated the metabolome analysis and increased the annotation rate. MN did not only reveal the structural relationships between the secondary metabolites, but also enabled a rapid annotation of related compounds in a cluster. Many clusters contained ions that could not be annotated, suggesting that they are putative novel compounds, revealing the enormous potential of sea foam-derived fungi. Among the sea foam-derived isolates studied, *Penicillium* sp. isolate 31.68F1B represented the most chemically diverse and bioactive strain, particularly against phytopathogens. This strain will be prioritized in our future natural product discovery efforts. Strains such as *Emericellopsis* and *Cladosporium,* which displayed a few distinct peaks in their UPLC metabolite profiles (Figure S4 and S5), may be good candidates for co-cultivation or one strain many compounds (OSMAC) experiments to enhance their chemical diversity and increase the amounts of bioactive metabolites [61]. Hence, sea foam-associated fungi appear to have a promising potential for the discovery of new lead compounds for agricultural and pharmaceutical applications.

## 4. Materials and Methods 

### 4.1. Fungal Materials

The fungal strains used in this study were isolated from a foam sampled from the Baltic Sea environment, Windebyer Noor in Schleswig-Holstein, Germany [12]. The isolates were identified based on the morphology and molecular analysis of the ITS1-5.8S-ITS2 regions and 18S rRNA gene whenever the amplification of the ITS region failed. The six strains were maintained on PDA medium (potato starch 4 g, glucose monohydrate 4 g, agar 15 g, 1000 mL H_2_O), and also cryo-conserved using the Microbank system (PRO-LAB Diagnostics, Richmond Hill, Ontario, Canada). 

### 4.2. Phylogenetic Analyses

ITS and one 18S rRNA gene sequence of the strains were aligned to their 10 respective closest relatives, according to two different, newly-conducted BLAST searches, (i) all highly similar sequences, and (ii) highly similar sequences from type material. Alignments were generated using the ClustalW option of the BioEdit sequence alignment editor [66]. The maximum likelihood method was applied for phylogenetic tree calculation using the GTR model. Phylogenetic calculations were performed using the MEGA7 platform [67]. The bootstrap method was used for the test of phylogeny, which was generated with 100 replicates.

### 4.3. Fermentation and Extraction

Fungal cultures were maintained on PDA at 22 °C for 14 days as pre-cultures. Pre-cultures were separately inoculated onto PDA in triplicates and cultivated for 21 days at 22 °C in the dark. The cultures were extracted with EtOAc in a 1:4 (agar:solvent) ratio. To achieve the highest extraction efficiency, agar cultures were first sliced and homogenized with an ultra-turrax in the extracting solvent. The EtOAc extracts were sequentially washed with an equal volume of Milli-Q^®^ (Arium^®^, Sartorius) water in a liquid–liquid partitioning experiment to deplete salts from the extracts. The clearly separated EtOAc layer was then evaporated to dryness. Dried extracts were solubilized in MeOH and filtered (0.2 μm filter) into storage vials and dried in vacuo. Aliquots of 1 mg were transferred into separate vials for bioactivity testing and UPLC-MS/MS profiling. 

### 4.4. UPLC/ESI-QToF-MS/MS Analyses

UPLC–MS analysis of the crude extracts (concentration 1 mg/mL) were performed on an Acquity UPLC I-Class System coupled to a Xevo G2-XS QToF Mass Spectrometer (Waters, Milford, MA, United States) controlled by MassLynx version 4.1. Samples were injected and separated on an Acquity UPLC HSS T3 column (High Strength Silica C18, 1.8 mm, 100 × 2.1 mm, Waters, Milford, MA, United States) at a temperature of 40 °C with an injection volume of 0.2 µL. A binary mobile phase system comprised of mobile phase A: 99.9% MilliQ®-water / 0.1% formic acid (ULC/MS grade) and mobile phase B: 99.9% acetonitrile (MeCN, ULC/MS grade, Biosolve BV, Dieuze, France)/0.1% formic acid. They were pumped at a rate of 0.6 mL/min with a linear gradient starting with 99% A from minute 0–11.5, followed by 0% A for 1 min (11.5–12.5), and back to the starting condition for 2.5 minutes. MS was done with an electrospray ionization source over a mass range of *m/z* 50–1600 Da in the positive mode with a capillary voltage of 0.8 kV, cone gas flow of 50 L/h, desolvation gas flow of 1200 L/h, source temperature of 150 °C, desolvation temperature of 550 °C with sampling cone and source offset at 40 and 80, respectively. The MS/MS experiments were carried out in tandem with ramp collision energy (CE): Low CE from 6 to 60 eV and a high CE of 9 to 80 eV. Solvents and PDA medium extracts were also analyzed.

### 4.5. Data Processing and Metabolomics

#### 4.5.1. Molecular Networking

UPLC–MS/MS data were converted to mzXML format, which were then uploaded to the Global Natural Products Social (GNPS) MN webserver, and analyzed using the MN workflow published by Wang et al. (2016) [68]. The data was filtered by removing all MS/MS peaks within +/- 17 Da of the precursor m/z. The MS/MS spectra were window filtered by choosing only the top six peaks in the +/- 50 Da window throughout the spectrum. The data was then clustered with MS-Cluster with a parent mass tolerance of 0.04 Da and a MS/MS fragment ion tolerance of 0.04 Da to create consensus spectra. Further, consensus spectra that contained less than two spectra were discarded. A network was then created, where edges were filtered to have a cosine score above 0.7, and more than six matched peaks. Further edges between two nodes were kept in the network if and only if each of the nodes appeared in each other’s respective top 10 most similar nodes. The spectra in the network were then searched against GNPS’ spectral libraries. The library spectra were filtered in the same manner as the input data. All matches kept between network spectra and library spectra were required to have a score above 0.7, and at least six matched peaks. Generated networks were visualized using Cytoscape version 3.6.14 [69], where nodes corresponding to peaks originating from media, and solvents were deleted. However, MN may contain nodes representing adducts and isotopic peaks of some parent ions. Molecular formula predictions were done with MassLynx version 4.1. for the annotation of parent ions. Predicted molecular formulae were searched against databases, such as the Dictionary of Natural Product (DNP), MarinLit, Reaxys and SciFinder. Dereplicated peak ions were further affirmed by comparing the experimental fragments to in-silico fragments generated from the CFM-ID web server [70] and the biological source of the hit. 

The molecular networking job on GNPS can be found at https://gnps.ucsd.edu/ProteoSAFe/status.jsp?task=e0941ddad1494f339e4b9a8977f6541a for the three *Penicillium* sp.; https://gnps.ucsd.edu/ProteoSAFe/status.jsp?task=93858c35393a4eea8b0c3d5500e7aac3 for *Plectospaerella* sp. and https://gnps.ucsd.edu/ProteoSAFe/status.jsp?task=3d38d5ea53fb429e9bbd99204b0d9bcc for *Cladosporium* sp. and *Emericellopsis* sp.

#### 4.5.2. Statistical Analysis

UPLC-MS/MS data (triplicate) of all fungi were converted to mzXML files and uploaded into Progenesis QI software (Nonlinear Dynamics, Version 3.0.1, Milford, United States, http://www.nonlinear.com/progenesis/qi/), for pretreatment. The adduct ions [M + H − H_2_O]^+^, [M + H]^+^, [M + Na]^+^, [M + K]^+^, [2M + H]^+^, [2M + Na]^+^ and [2M + K]^+^ were selected based on the observed ionization behavior. Alignment was done automatically. Peak picking was done with a retention time limit of 11.30 mins. The default noise algorithm was adopted with sensitivity set to automatic. Subsequently, deconvolution and peak grouping was done. The extracted metabolic features were exported to EZinfo 3.0 (an extension of Progenesis) for statistical analysis and principal component analysis. PCA and loadings plots were generated in EZinfo 3.0 using the PLS-DA model with Pareto scaling.

### 4.6. Antimicrobial Activity

Crude extracts were tested for inhibitory activity against human and economically relevant crop pathogens. These test microbes were cultivated and maintained on their respective optimal media. Bioactivity testing was performed in 96-well plates with an effective concentration of 100 µg/mL of crude extracts and 0.5% *v*/*v* dimethyl sulfoxide (DMSO). Crude extracts were prepared as 20 mg/mL stock solutions in DMSO, and serially diluted with medium (test microbe specific), and introduced onto the test pathogens in the well plates. Bacterial and yeast cells were grown overnight in liquid media at 28 °C (37 °C for *E. faecium*) with shaking (160 rpm) and subsequently diluted with media to obtain specific optical densities (test microbe-specific) as loading inocula. These inocula were added to the crude extracts in the well plates to a final volume of 200 µL and then incubated at 200 rpm (No shaking with *E. faecium* test). Correspondingly, the antifungal activity of the crude extracts was assessed using 10^4^ spores/mL of fungal pathogen. Pathogenic fungi were grown on specific media for 14 days. Spores were collected and diluted in specific broth media and then added to the crude extracts in microplates, which were incubated at 200 rpm. Incubation times and temperatures are specific to test microbes. All samples were tested in triplicates, including positive controls (specific to test microbe) and negative controls, which constituted inocula without crude extract and 0.5% DMSO. With the exception of the *E. faecium* test, which employed bromocresol purple, the viability of bacterial pathogens after inoculation was assessed by adding 10 µL of resazurin solution (0.3 mg/mL phosphate-buffered saline) and measuring the fluorescence at 560 nm using an Infinite M200 plate reader (TECAN Deutschland GmbH, Crailsheim, Germany). For fungal tests, absorbances at 600 nm before and after incubation were measured. Bioactivities of crude extracts were quantified in IC_50_ values using MS Excel. Test pathogens and their specific conditions for the bioactivity protocol above are detailed in Table S4.

### 4.7. Anticancer Activity

The anticancer and cytotoxicity of the crude extracts (at a concentration of 100 µg/mL) were assessed using the CellTiterBlue Cell Viability Assay (Promega, Mannheim, Germany) against A-549 lung carcinoma cells, MDA-MB-231 breast cancer cells and HaCaT human keratinocytes (Cell Line Service, Eppelheim, Germany). The cells were seeded into 96-well microplates at a concentration of 1 × 10^4^ cells /well. They were cultivated in RPMI medium augmented with 10% fetal bovine serum, 100 µg/mL penicillin, and 100 mg/mL streptomycin at 37 °C under a humidified atmosphere and 5% CO_2_. 

After 24 hrs of cultivation, the medium was replaced with fresh medium (100 µl) containing the crude extracts, and again cultivated for 24 hrs. Further tests were done according to the CellTiterBlue Cell Viability Assay protocol described by the manufacturer (Promega, Mannheim, Germany). The anticancer drug tamoxifen was used as the positive control. The growth medium and 0.5% DMSO were tested as negative controls. The inhibition rates were computed from fluorescence measurements taken with the microplate reader, with excitation and emission wavelengths of 560 nm and 590 nm, respectively. 

## Figures and Tables

**Table 1:** Selected fungi and their GenBank accession numbers.

No.	Isolate	Fungal ID	Accession Number
**1**	B2F1B	*Plectosphaerella* sp.	MH791266.1
**2**	86F1C	*Cladosporium* sp.	MH791259.1
**3**	25.88F1C	*Emericellopsis* sp.	MH791280.1
**4**	36.97F1C	*Penicillium* sp.	MH791183.1
**5**	31.68F1B	*Penicillium* sp.	MH791236.1
**6**	62.72F1A	*Penicillium* sp.	MH791282.1

**Table 2:** Dereplication table of highlighted metabolites in the PCA loadings plot, unique to the individual *Penicillium* strains and contributing to the discrimination in the PCA plot. M represents neutral mass.

Source	t*_R_* (min)	*m/z*	Mol. formula for M	[ppm] difference	Identification
*Penicillium* sp. 31.68F1B	6	207.0678 [M + H]^+^	C_11_H_10_O_4_	0.0	7-hydroxy-2-(hydroxymethyl)-5-methyl-4H-1-benzopyran-4-one
	8.2	238.1236 [M + H]^+^	C_16_H_15_NO	2.5	n.d.
	6	685.2246 [2M + H]^+^	C_13_H_18_N_4_O_7_	0.6	n.d.
*Penicillium* sp. 36.97F1C	2.5	209.0474 [M + H]^+^	C_10_H_8_O_5_	1.9	viticolin C
	4.8	368.1857 [M + H]^+^	C_21_H_25_N_3_O_3_	0.5	brevianamide E
	4.7	359.1101 [M + Na]^+^	C_17_H_20_O_7_	1.7	4-hydroxymycophenolic acid
*Penicillium* sp. 62.72F1A	3.8	309.1338 [M − H_2_O + H]^+^	C_16_H_22_O_7_	1.5	n.d.
	5	390.1927 [M + H]^+^	C_22_H_23_N_5_O_2_	0.5	tryhistatin
	5.7	293.1035 [M + H]^+^	C_15_H_16_O_6_	5.0	italicic acid

n.d.: not determined or no hit from microbial natural product databases.

**Table 3:** The IC_50_ values (in µg/mL) of the active fungal extracts against highly susceptible (>70% inhibition at 100 µg/mL concentration) plant and human pathogens. Pi, *P. infestans*; Mo, *M. oryzae*; Bc, *B. cinerea;* Efm, *E. faecium*; MRSA, methicillin-resistant *S*. *aureus*; Ca, *C. albicans*; Cn, *C. neoformans*. Positive controls: cycloheximide for Pi and boscalid for Bc; nystatin for Ca and Mo; ampicillin for Efm; chloramphenicol for MRSA; amphotericin B for Cn.

Fungus	Plant Pathogens			Human Pathogens			
	Pi	Mo	Bc	Efm	MRSA	Ca	Cn
31.68F1B	4.3	2.2	13.8	15.0	31.0	6.3	9.2
36.97F1C	31.6	12.9	>100	37.8	>100	>100	26.9
Positive control	0.1	0.4	0.1	1.7	0.5	1.1	0.6

## Supplementary Materials

The following are available online at https://www.mdpi.com/1660-3397/18/2/128/s1, Figure S1: Phylogenetic analysis of selected foam-derived fungal strains based on ITS region; Figure S2: Phylogenetic analysis of selected strain *Penicillium* sp. isolate 36.97 F1C based on the 18S rRNA gene; Figure S3: Experimental and library MS/MS spectra of mycophenolic acid; Figure S4: Base peak chromatograms of the extracts of selected foam-derived fungal strains; Figure S5: Chemical structures of metabolites annotated in the selected sea foam-derived fungi; Table S1: Identification of six foam-derived fungal strains isolated from Windebyer Noor (Nov, 2015), according to the two new BLAST searches; Table S2: Putative identification of metabolites produced by foam-derived fungi; Table S3: Bioactivity (% inhibition at 100 µg /ml concentration) of the foam-derived fungi; Table S4: Test pathogens and their specific conditions for bioactivity testing.
