# Supplementary material for: Mining the Metabolome and the Agricultural and Pharmaceutical Potential of Sea Foam-Derived Fungi
Source: Mar Drugs. 2020 Feb 22;18(2):128. doi: 10.3390/md18020128 (PMC7074149; doi:10.3390/md18020128)
Supplement: Supplementary file 1 [file marinedrugs-18-00128-s001.pdf]

Supplementary materials

Mining the Metabolome and the Agricultural and Pharmaceutical Potential of Sea Foam-Derived Fungi

Ernest Oppong-Danquah [1], Cristina Passaretti [1], Orazio Chianese [1], Martina Blümel [1] and Deniz Tasdemir [1, 2*]

[1] GEOMAR Centre for Marine Biotechnology (GEOMAR-Biotech), Research Unit Marine Natural Products Chemistry, GEOMAR Helmholtz Centre for Ocean Research Kiel, Am Kiel-Kanal 44, 24106 Kiel, Germany; eoppong-danquah@geomar.de (E.O.-D.); cr.passaretti92@gmail.com (C.P.); ory_oi91@hotmail.it (O.C.); mbluemel@geomar.de (M.B.)

[2] Faculty of Mathematics and Natural Science, Kiel University, Christian-Albrechts-Platz 4, 24118 Kiel, Germany

* Correspondence: dtasdemir@geomar.de; Tel.: +49-431-6004430; **ORCID ID:** 0000-0002-7841-6271

List of Tables **Page No.**

Table S1. Identification of six foam-derived fungal strains isolated from Windebyer Noor (Nov. 2015) according to the two new BLAST searches 2

Table S2. Putative identification of metabolites produced by foam-derived fungi 5

Table S3: Bioactivity (% inhibition at 100 μg/ml concentration) of the foam-derived fungi 11

Table S4. Test pathogens and their specific conditions for bioactivity testing 12

List of Figures

Figure S1. Phylogenetic analysis of selected foam-derived fungal strains based on ITS region 14

Figure S2. Phylogenetic analysis of selected strain *Penicillium* sp. isolate 36.97 F1C based on 18S rRNA gene 15

Figure S3: Experimental and library MS/MS spectra of mycophenolic acid 15

Figure S4. Base peak chromatograms of extracts of selected foam-derived fungal strains 16

Figure S5. Chemical structures of metabolites annotated in the selected sea foam - derived fungi 17

Table S1. Identification of six foam-derived fungal strains isolated from Windebyer Noor (Nov, 2015) according to the two new BLAST searches.

Query		BLAST ALL			BLAST TYPE			
Isolate name	Acc. No	Name	Acc. No.	Similarity (%)	Name	Acc. No.	Similarity (%)	Taxonomic assignment
B2F1B	MH791266.1 *Plectosphaerella* sp.	Fungal sp. strain S255T	KU839553.1	99.79	*Plectosphaerella niemeijerarum* CBS 143233 T	NR_156677.1	99.59	*Plectosphaerella sp.*
						MG386080.1	99.59	
		Fungal sp. strain S255S	KU839552.1	99.79	*Plectosphaerella oligotrophica* CGMCC 3.15078 T	NR_155632.1	98.35	
		Fungal sp. strain S255O	KU839551.1	99.79	*Plectosphaerella oligotrophica* strain LC1990	JX508810.1	98.35	
		Fungal sp. strain S255N	KU839550.1	99.79	*Plectosphaerella delsorboi* CBS 116708T	NR_155694.1	96.30	
						KY499237.1	96.30	
		Fungal sp. strain S255L	KU839549.1	99.79	*Plectosphaerella populi* CBS 139623	NR_138002.1	95.08	
25.88F1C	MH791280.1 *Emericellopsis* sp.	*Emericellopsis* sp. s012	HQ649988.1	95.30	*Emericellopsis terricola* CBS 120.40 T	NR_159843.1	94.97	*Emericellopsis* sp.
						MH856058.1	94.97	
		Emericellopsis terricola CBS 120.40 T	NR_159843.1	94.97	*Acremonium tubakii* CBS 790.69	NR_163810.1	94.63	
						MH859429.1	94.63	
		Emericellopsis terricola strain CBS 126950	MH864349.1	94.97	*Emericellopsis microspora* strain CBS 380.62	MH858186.1	94.63	
		Emericellopsis terricola strain CBS 387.70A	MH859748.1	94.97	*Emericellopsis glabra* strain CBS 119.40	MH856057.1	93.55	
		Emericellopsis terricola strain CBS 246.70	MH859590.1	94.97	*Emericellopsis pusilla* CBS 226.62 T	NR_159878.1	94.30	

86F1C	MH791259.1 *Cladosporium cladosporioides*	*Cladosporium asperulatum* isolate LH4	MN220523.1	100	*Cladosporium verrucocladosporioides* strain CBS 126363	MH863939.1	100	*Cladosporium* sp.
		Cladosporium sp. strain SMY02	MK355726.1	100	*Cladosporium antarcticum* CBS 690.92 T	NR_121332.1	99.79	
						EF679334.2	99.79	
		Cladosporium sp. isolate 18MWF13.1	MN128233.1	100	*Cladosporium ossifragi* culture CBS:842.91	EF679381.2	99.79	
		Cladosporium cladosporioides isolate C11	MK813962.1	100	*Cladosporium iridis* strain CBS 138.40	EU167591.1	99.79	
		Cladosporium cladosporioides isolate C10	MK813961.1	100	*Cladosporium subinflatum* strain CBS 121630	MH863129.1	99.79	
31.68F1B	MH791236.1 *Penicillium bialowiezense*	*Penicillium* sp. isolate P11-16A	MN186768.1	100	*Penicillium bialowiezense* strain CBS 227.28 T	NR_111323.1	100	*Penicillium bialowiezense*
						MH854996.1		
		Penicillium sp. isolate P8-16A	MN186762.1	100	*Penicillium brunneostoloniferum* strain CBS 317.59	KC411734.1	98.77	
		Penicillium sp. isolate SL71_37c_D	MN105533.1	100	*Penicillium brevicompactum* NRRL 2011T	NR_121299.1	98.77	
						AY484912.1		
		Penicillium sp. isolate SL16_77a_G2	MN105477.1	100	*Penicillium brevicompactum* strain CBS 257.29	KF465776.1	98.77	
		Penicillium sp. isolate SL63_46_G	MN105342.1	100	*Penicillium patris-mei* CBS 210.28 T	NR_138315.1	98.46	
36.97F1C	MH791183.1 *Penicillium* sp.	*Penicillium* sp. FA6-2	KF776918.1	98.04	*Penicillium limosum* CBS 339.97 T	NG_062729.1	97.77	*Penicillium* sp.
						EF411061.1		

	**18S rRNA gene**	*Penicillium* sp. FA9	KF776917.1	98.04	*Penicillium commune* CBS 343.51 T	NG_062634.1<br>AF548088.1	97.77	
		Penicillium brevicompactum isolate PenC	FJ717699.1	98.04	*Talaromyces leycettanus* CBS 398.68T	NG_062664.1	97.68	
		Penicillium sp. strain OUCMDZ-4920	KY448996.1	98.04	*Talaromyces wortmannii* CBS 391.48T	NG_062801.1<br>GU733355.1	97.59	
		Penicillium brevicompactum strain KR5	MH047201.1	97.86	*Penicillium roqueforti* strain ATCC 10110	GQ458035.1	97.59	
62.72F1A	MH791282.1 *Penicillium brevicompactum*	*Penicillium* sp. isolate P11-16A	MN186768.1	100	*Penicillium bialowiezense* strain CBS 227.28 T	MH854996.1<br>NR_111323.1	100	*Penicillium bialowiezense*
		Penicillium sp. isolate P8-16A	MN186762.1	100	*Penicillium brunneostoloniferum* strain CBS 317.59	KC411734.1	98.62	
		Penicillium sp. isolate SL71_37c_D	MN105533.1	100	*Penicillium patris-mei* CBS 210.28 T	NR_138315.1	98.62	
		Penicillium sp. isolate SL16_77a_G2	MN105477.1	100	*Penicillium neocrassum* NRRL 35639 T	NR_121321.1	98.62	
		Penicillium sp. isolate SL63_46_G	MN105342.1	100	*Penicillium brevicompactum* NRRL 2011	NR_121299.1	98.62	

Table S2. Putative identification of metabolites produced by foam-derived fungi. Annotations were made based on *m/z* $[M+H]^+$ or other adducts (specified), biological source, retention time (t_R), predicted molecular formula and fragmentation pattern. Confidence level of putative identification after Sumner et al. [1].

Compound I.D	t_R	Parent mass $[M+H]^+$	Putative ID	Chemical class/ family	Molecular formula *m/z* $[M+H]^+$	Δ ppm	MS^2	Confidence level	Isolate	Reference
1	0.6	293.1001	italicic acid	polyketide	$C_{15}H_{17}O_6$	5.0	281.0570; 267.0149; 253.0010; 237.0129; 202.0589; 187.9807; 163.0728; 145.0589; 127.0410; 118.0874; 99.0874; 81.0292	3	62.72F1A	[2]
2	3.7	235.0962	citreopyrone E	pyranone	$C_{13}H_{15}O_4$	-3.4	217.0848; 191.0696; 179.1068; 151.0409; 135.0788; 126.1260; 112.9818; 86.0965; 67.0180	3	31.68F1B	[3]
3	3.9	271.1441	quinolactacin A1	alkaloid	$C_{16}H_{19}N_2O_2$	-1.1	214.0740; 233.0798; 203.1697; 189.9731; 185.4548; 171.0642; 161.0518; 146.8997; 129.0546; 123.1155; 112.9648; 97.0940; 86.0966; 84.9576	3	31.68F1B	[4,5]
4	5.9	445.2588	preaustinoid A	meroterpenoid	$C_{26}H_{37}O_6$	-0.7	385.2373; 413.2239; 367.2273; 243.1746	2	31.68F1B, 36.97F1C, 62.72F1A	[6,7]
5	5.2	461.2432	preaustinoid A1	meroterpenoid	$C_{26}H_{37}O_7$	-2.0	443.2457; 411.2151; 383.2230, 365.2193; 213.1639	3	31.68F1B, 62.72F1A	[7]

6	6.0	207.0687	7-Hydroxy-2-(hydroxymeth5-methyl-4H-1-benzopyran-4-one	benzopyranone	$C_{11}H_{11}O_4$	0	189.0543; 177.0549; 151.0384; 131.0485; 117.0320; 53.0011; 77.0384; 91.0539	3	31.68F1B	[8]
7	6.0	321.1381	mycophenolic acid	meroterpenoid	$C_{17}H_{21}O_6$	2.5	303.1240; 275.1284; 285.1128; 207.0660; 195.0655; 159.0447; 177.0548; 159.0447; 109.0644; 81.0686; 77.0412; 69.9658	2	31.68F1B, 62.72F1A, 36.97F1C	[9,10]
8	4.7	359.1101 [M+Na]	4-hydroxy-mycophenolic acid	meroterpenoid	$C_{17}H_{20}O_7Na$	1.7	-	2	36.97F1C,	[9,10]
9	7.5	335.1489	mycophenolic acid methyl ester	meroterpenoid	$C_{18}H_{23}O6$	-1.8	303.1239; 275.1292; 207.0660	2	31.68F1B, 36.97F1C	[10]
10	6.3	582.31	nodulisporic acid D_3	indole-diterpenoid	$C_{38}H_{48}NO_4$	-4.1	570.3046; 564.3406; 536.3499; 519.3196; 483.2853; 453.3004; 385.1725; 367.1622; 349.1508; 253.1283; 202.0804; 170.1904; 60.0423	3	31.68F1B	[11]
11	6.0	179.0743	7-Hydroxy-4,6-dimethyl-1(3*H*)-isobenzofuranone	polyketide	$C_{10}H_{11}O_3$	-3.4	-	3	31.68F1B , 62.72F1A, 36.97F1C	[12,13]
12	6.7	211.0971	canadensolide	polyketide	$C_{11}H_{15}O_4$	0	193.0877; 175.0769; 165.0925; 141.0197	3	62.72F1A	[14,15]

13	7.1	523.2237	asperphenamate analogue; Tyr sub Phe	phenylalanine derivatives	$C_{32}H_{30}N_2O_5$	1.0	505.3368; 256.1347; 238.1242; 134.0967	2	31.68F1B, 62.72F1A, 36.97F1C	[16]
14	7.5	259.0606	alternariol	benzocoumarin	$C_{14}H_{11}O_5$	0	-	3	31.68F1B	[17]
15	7.5	607.1083	xanthoepocin	polyketide	$C_{30}H_{23}O_{14}$	0	589.0986; 571.0874; 511.0656; 469.0561; 380.0502; 313.0381; 287.0562; 275.0553; 259.0594; 243.0286; 75.7144	2	31.68F1B	[18]
16	7.5	487.2709	andrastin A	meroterpenoid	$C_{28}H_{39}O_7$	-2.1	427.2476; 409.2382; 395.2220; 377.2119; 243.1754; 183.0657	2	31.68F1B	[19]
17	7.6	489.2671	andrastin B	meroterpenoid	$C_{28}H_{41}O_7$	1.7	449.2322; 428.2501; 410.2356; 396.2200; 243.1831; 225.1616; 183.0701	3	31.68F1B	[19]
18	7.7	429.2430	andrastin D	meroterpenoid	$C_{26}H_{37}O_5$	1.0	410.2400; 396.2190; 378.2111; 244.1802; 151.0384	3	31.68F1B	[19]
19	7.7	519.1899	rubratoxin B	nanodride polyketide	$C_{26}H_{31}O_{11}$	4.4	292.0955; 282.0752; 250.0851	3	31.68F1B, 36.97F1C	[20]
20	7.7	625.3936	citrinadin A	indole alkaloid	$C_{35}H_{53}N_4O_6$	-3.9	593.0722; 551.0601; 367.2100; 281.1600	3	31.68F1B	[21]
21	8.2	507.232	asperphenamate	phenylalanine derivative	$C_{32}H_{31}N_2O_4$	0.4	377.3205; 256.1341; 238.1238; 224.1077; 134.0963	2	31.68F1B, 36.97F1C, 62.72F1A	[16]

22	4.9	366.1812	brevianamide A	indole alkaloid	$C_{21}H_{24}N_3O_3$	3.5	348.1708; 338.1867; 321.1603; 235.1445; 176.1068; 54.0934	3	62.72F1A, 36.97F1C	[22]
23	5.1	390.18	roquefortine C	diketopiperazine	$C_{22}H_{24}N_5O_2$	-0.3	322.1290; 193.0723	3	62.72F1A	[23]
24	7.7	493.2815	fungisporin:<br>cyclo-(Phe-Phe-Val-Val)	cyclic tetrapeptide	$C_{28}H_{37}N_4O_4$	0.6	465;2788; 394.2130; 346.2133; 295.1447; 247.1454; 219.1501; 199.1445; 120.0810	2	62.72F1A	[24]
25	6.5	509.2759	cyclo-(Tyr-Phe-Val-Val)	cyclic tetrapeptide	$C_{28}H_{37}N_4O_5$	-1.0	410.2088; 365.1879; 346.2130; 311.1393; 283.1457; 247.1457; 219.1501; 199.1463; 136.0762; 120.0913	2	62.72F1A, 36.97F1C	[24]
26	7.4	532	cyclo-(Phe-Trp-Val-Val)	cyclic tetrapeptide	$C_{30}H_{38}N_5O_4$	-0.6	504.2950; 433.2282; 385.2233; 334.1559; 286.1519; 258.1613; 247.1449; 199.1477; 159.0923; 120.0829	2	62.72F1A, 31.68F1B	[24]
27	6.9	562	cyclo-(Tyr-Trp-Val-Ile)	cyclic tetrapeptide	$C_{31}H_{40}N_5O_5$	-0.2	534.3136; 463.2361; 449.2158; 399.2369; 350.1498; 322.1569; 300.1726; 286.1556; 258.1624; 213.1596; 159.0920; 136.0750	2	31.68F1B	[24]
28	7.5	575.0833	xanthomegnin	binaphthoquinone	$C_{30}H_{23}O_{12}$	22*	557.0644; 529.0737; 497.0429; 459.0681; 243.0282	3	36.97F1C, 31.68F1B	[25]
29	6.5	471.2722	communesin G	indole alkaloid	$C_{29}H_{35}N_4O_2$	-6.8	439.2459; 261.1839; 233.0794	3	31.68F1B	[26]

30	2.5	209.0474	viticolin C	tropolone	$C_{10}H_9O_5$	1.9	191.0344; 181.0497; 163.0390; 147.0440	3	36.97F1C	[27]
31	6.2	446.1970	fumiquinazoline A	imidazoindole	$C_{24}H_{24}N_5O_4$	10	228.1018; 200.1068; 132.0487; 175.0364	3	36.97F1C, 62.72F1A	[28]
32	5.7	596.3093	JBIR-113	cyclodespsipeptide	$C_{31}H_{42}N_5O_7$	1.5	485.2384; 467.2200; 409.2340; 356.1597; 338.2077; 280.1658; 209.1296; 140.0705	2	36.97F1C	[29]
33	10.7	263.2365 (M+H-H_2O)	conjugated linoleic acid (9E,11E) - primary metabolite	fatty acid	$C_{18}H_{31}O$	2.3	245.2311; 189.1600; 175.1515; 163.1522; 149.1302; 123.1240	3	36.97F1C, 31.68F1B	GNPS library
34	4.8	368.1857	brevianamide E	indole alkaloid	$C_{21}H_{26}N_3O_3$	0.5	348.1715; 338.1873; 321.1608; 310.1924; 235.1451; 176.1075	3	36.97F1C	[22]
35	5	390.1927	tryhistatin	diketopiperazine	$C_{22}H_{24}N_5O_2$	0.5	322.1304; 193.0731	3	62.72F1A	[30]
36	1.5	173.0570	4-(3-furanyl)-benzaldehyde	polyketide	$C_{11}H_8O_2$	11*	130.9707; 112.1531; 96.9574	3	86F1C, 25.88F1C	[31]
37	2.4	167.0703	2′,4′-dihydroxy-3′-methylacetophenone	polyketide	$C_9H_{11}O_3$	-1.2	149.0591; 137.9860; 130.0076; 125.0595; 114.9765; 108.9610; 96.9611; 79.0490	3	86F1C, 25.88F1C	[32]
38	7.7	515.2657	lucidenic acid D	triterpenoid	$C_{29}H_{39}O_8$	-1.0	419.2328; 392.1592; 363.1694; 307.1118; 216.9408; 184.0787; 172.1043;	3	86F1C, 25.88F1C	[33]
39	11.2	475.2885	methyl lucidenate N	triterpenoid	$C_{28}H_{43}O_6$	-14.3*	419.2301; 363.1702; 307.1100; 250.1831	3	86F1C, 25.88F1C	[33]

40	9.0	791.2353	cladochrome D	perylenequinone	$C_{44}H_{39}O_{14}$	0.5	663.1892; 637.2188; 601.2106; 552.1516; 515.1664; 483.1454; 473.1285; 443.1058; 404.1078; 359.6608; 317.2110; 301.5874; 286.8502; 253.0100; 195.1283; 163.0770; 105.0340;	3	86F1C	[34]
41	12.3	593.4031	5α,8α-epidioxy-24(R)-methylcholesta-6,22-dien-3β-D-glucopyranoside	sterol	$C_{34}H_{57}O_{8}$	-5.1	495.2724; 451.3066; 393.1087; 355.0716; 307.1878; 251.0862; 223.0799; 165.0840; 147.0609	3	86F1C	[35]
42	7.7	493.2807	preaustinoid D	meroterpenoid	$C_{27}H_{41}O_{8}$	1.2	394.2118; 346.2113; 295.1448; 267.1487; 247.1447; 219.1497; 120.0802	3	86F1C, 25.88F1C	[36]
43	12.3	685.4373	icosalide A3	cyclic peptide	$C_{34}H_{61}N_{4}O_{10}$	2.9	555.2990; 527.1161; 478.3198; 442.3124; 378.3267; 341.0226; 195.1078; 133.0927	3	86F1C, 25.88F1C	[27]
44	7.5	470.3380	cytochalasin R	indole alkaloid	$C_{28}H_{40}NO_{5}$	18	452.3407; 236.1507	3	B2F1D	[37]
45	7.8	279.0750	betulinan C	pyranone	$C_{18}H_{15}O_{3}$	-29.7*	219.9331; 195.9081; 149.0201	3	B2F1D	[38]
46	1.8	184.0530	3-acetyl-5-isopropyl-pyrrolidine-2,4-dione	pyrrolidine alkaloid	$C_{9}H_{14}NO_{3}$	11	142.9600; 98.9577	3	B2F1D	[39]
47	9.8	444.2747	stachybotrin A	alkaloid	$C_{26}H_{38}NO_{5}$	0.0	426.2615; 416.2809; 402.2657; 360.1798;	3	B2F1D	[40]

							334.1642; 233.2270; 226.1070; 212.0551; 194.0448; 175.1481			
48	4.7	235.0969	alternariphent A1	phenol derivative	$C_{13}H_{15}O_4$	0.9	207.1034; 194.9547; 191.1058; 163.1119; 154.9656; 86.0951	3	B2F1D	[41,42]
49	6.6	583.3277	6-O-propionyl-6,16-O-dideacetylhelvolic acid	nortriterpenoid	$C_{34}H_{47}O_8$	0.0	543.1447; 421.2726; 391.2717; 281.1542; 229.1223; 123.1101	3	B2F1D	[43]

*Peak ions of low intensity (molecular formula prediction with high ppm errors)

Table S3: Bioactivity (% inhibition at 100 μg /ml concentration) of the foam-derived fungi. Mean values are based on triplicate measurements. Plant pathogens: Pss, *P. syringae*; Xc, *X. campestris*, Ea, *E. amylovora*; Rs, *R. solanacearum*; Pi, *P. infestans*; Mo. *M. oryzae*; Bc. *B. cinerea.* Human pathogens include the ESKAPE panel Efm, *E. faecium*; MRSA, methicillin-resistant *S. aureus*; Kp, *K. Pneumoniae*; Ab, *A. baumannii*; Psa, *P. aeruginosa*; Ec, *E. coli*; Ca, *C. albicans*; Cn, *C. neoformans*. 0.5% DMSO was used as a solvent control. (+) control see Table S4.

Sea foam derived fungus	Anti-microbial activity against plant pathogens							Anti-microbial activity against human pathogens									Anti-cancer Activity		
	Anti-bacterial				Anti-fungal			Anti-ESKAPE							Anti-fungal				
	Pss	Xc	Ea	Rs	Pi	Mo	Bc	Efm	MRSA	Kp	Ab	Psa	Ec	Ca	Cn	HaCaT	A549	MB231
Plectospaerella sp.	-	77	-	-	44	-	-	-	50	-	-	-	-	-	-	-	-	-
Cladosporium sp.	-	67	25	-	-	-	-	-	54	-	-	-	-	-	-	-	-	-
Emericellopsis sp.	-	42	-	-	-	-	-	-	37	-	-	-	-	-	-	-	-	-
Penicillium:31.68F1B	-	47	27	-	97	98	74	99	99	-	-	-	-	80	100	-	-	-

Penicillium:36.97F1C	-	29	-	-	86	100	41	100	33	-	20	-	-	66	82	-	-	-
Penicillium:62.72F1A	-	33	-	-	80	-	30	-	-	-	-	-	-	-	-	-	-	26
(+) control	95	95	66	99	96	97	98	99	93	99	96	99	88	85	100	41	32	62
(-) control (solv./PDA)	-	-	-	-	-	-	-	-	-	-	-	-	-	-	-	-	-	-

Table S4. Test pathogens and their specific conditions for bioactivity testing

**Human pathogen**	**Strain**	**Medium**	**Optical density**	**Preculture incubation temperature (°C)**	**Positive control**	**Incubation temperature (°C)**	**Incubation time (h)**
Enterococcus faecium	DSM 20478	M92	0.01	37	Ampicillin	37	4
Methicillin-resistant *Staphylococcus aureus* (MRSA)	DSM 18827	TSB12	0.01	28	Chloramphenicol	37	5
Klebsiella pneumoniae	DSM 30104	TSB12	0.01	28	Chloramphenicol	37	5
Acinetobacter baumannii	DSM 30007	TSB12	0.01	28	Ampicillin	37	5

Pseudomonas aeruginosa	DSM 1128	TSB12	0.01	37	Chloramphenicol	37	5
Escherichia coli	DSM 1576	TSB12	0.01	28	Chloramphenicol	37	5
Candida albicans	DSM 1386	M186	0.03	28	Nystatin	37	5
Cryptococcus neoformans	DSM 6973	M186	0.03	28	Amphotericin B	28	6-7
**Plant pathogens**							
Pseudomonas syringae pv. *aptata*	DSM 50252	TSB12	0.03	28	Chloramphenicol	28	9
Xanthomonas campestris	DSM 2405	TSB12	0.03	28	Chloramphenicol	28	7
Erwinia amylovora	DSM 50901	TSB12	0.03	28	Chloramphenicol	28	9
Ralstonia solanacearum	DSM 9544	M1	0.03	28	Tetracycline	28	9
Phytophthora infestans	CBS 120920	PB*	(10^4 spores)	22	Cycloheximide	22	74-96
Magnaporthe oryzae	DSM 62938	SA*	(10^4 spores)	22	Nystatin	22	74-96
Botrytis cinerea	DSM 5145	MA	(5×10^4 spores)	22	Boscalid	25	72

M92: 30 g tryptic soy broth, 3 g yeast extract, 1000 mL H_2O, pH 7.0-7.2
TSB12: 12 g tryptic soy broth, 5 g NaCl, 1000 mL H_2O
M186: 10 g glucose, 5 g peptone from soybeans, 3 g yeast extract, 3 g malt extract, 1000 mL H_2O
M1: 5 g peptone, 3 g peptone from meat, 1000 mL H_2O
PB: 150 g pea, 5 g glucose, 0.1 mg thiamine HCl, 1000 mL H_2O
SA: 10 g peptone, 20 g glucose, 15 g agar, 1000 mL H_2O, pH 5.6 ± 0.02
MA: 10 g malt extract, 15 g agar, 1000 mL H_2O
*: In addition to broth, 15 g agar was added for pre-cultivation of fungi

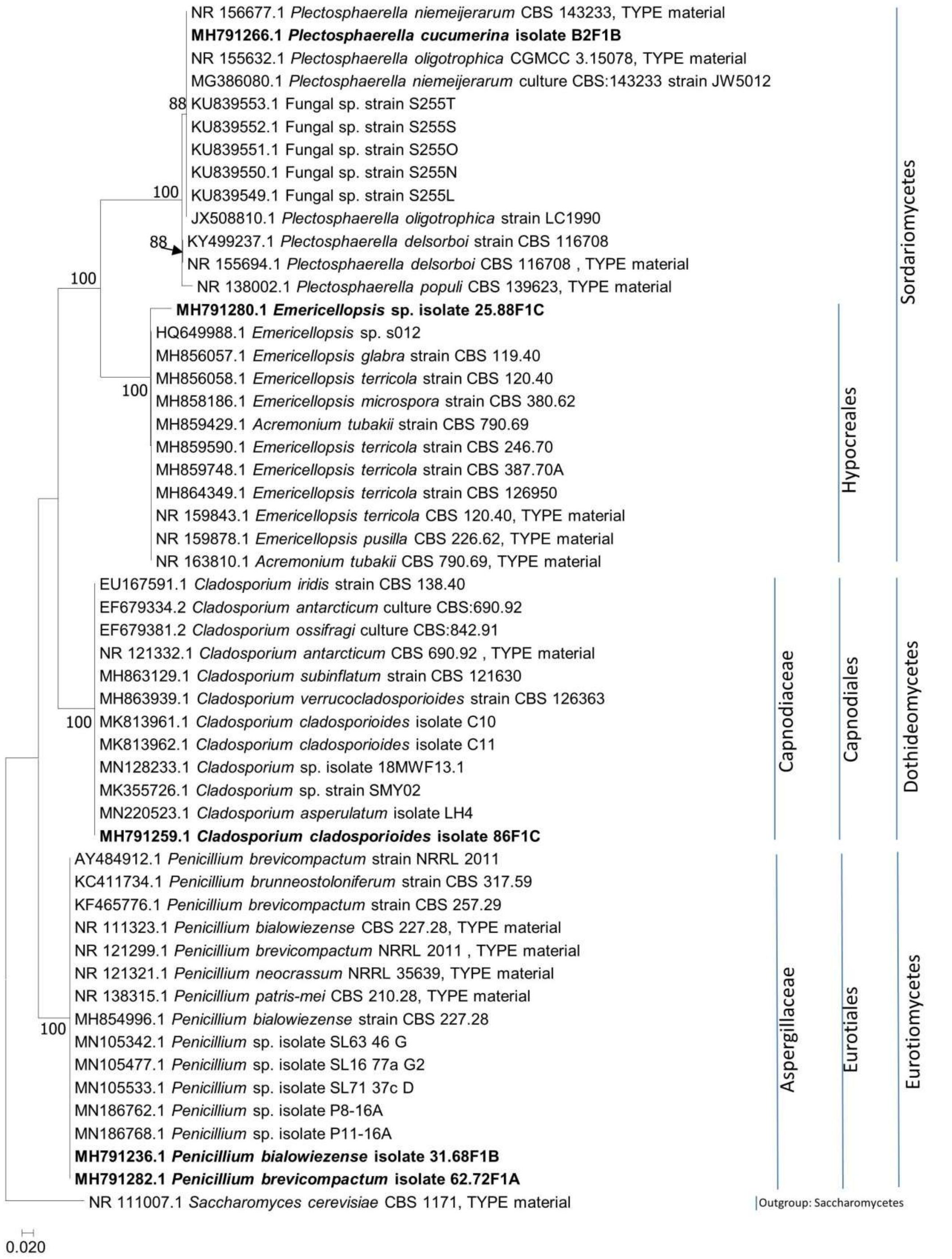

Figure S1. Phylogenetic analysis of selected foam-derived fungal strains based on ITS region. Reference sequences were downloaded from the NCBI database. *Penicillium* sp. isolate 36.97 F1C (only identified by 18S rRNA) is located in another phylogenetic tree (Figure S2).

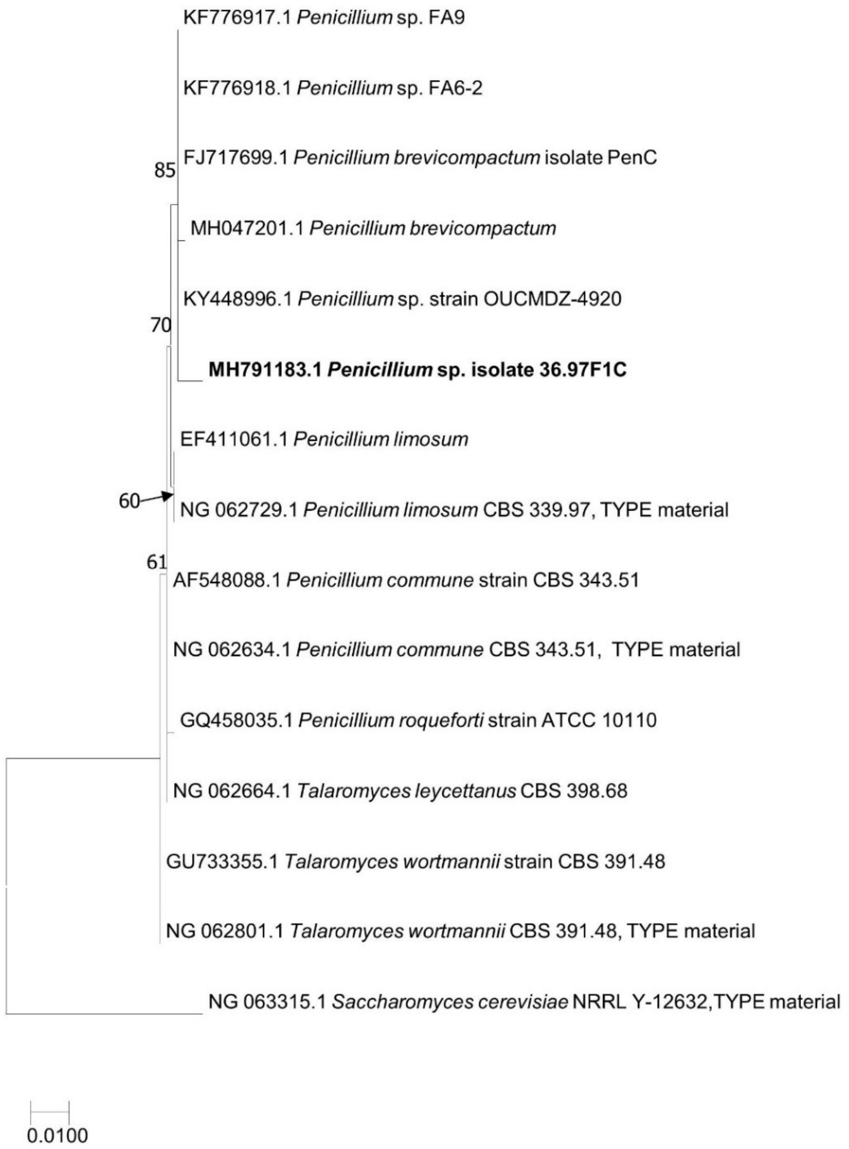

Figure S2. Phylogenetic analysis of selected strain *Penicillium* sp. isolate 36.97 F1C based on 18SrRNA gene. Reference sequences were downloaded from the NCBI database.

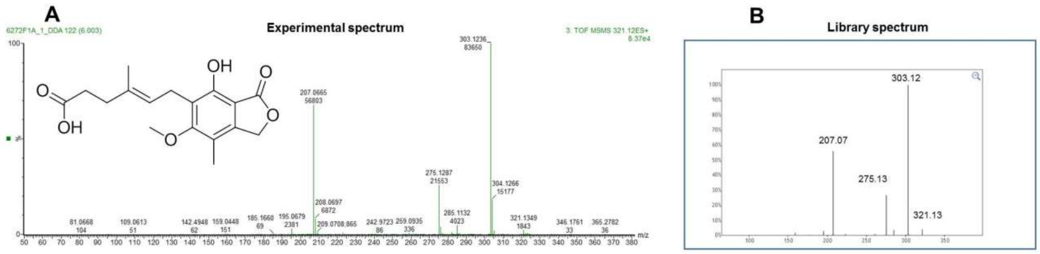

Figure S3: MS/MS spectrum of **(A)** node *m/z* $[M+H]^+$ 321.1342 annotated as mycophenolic acid and **(B)** library MS/MS spectrum of mycophenolic acid.

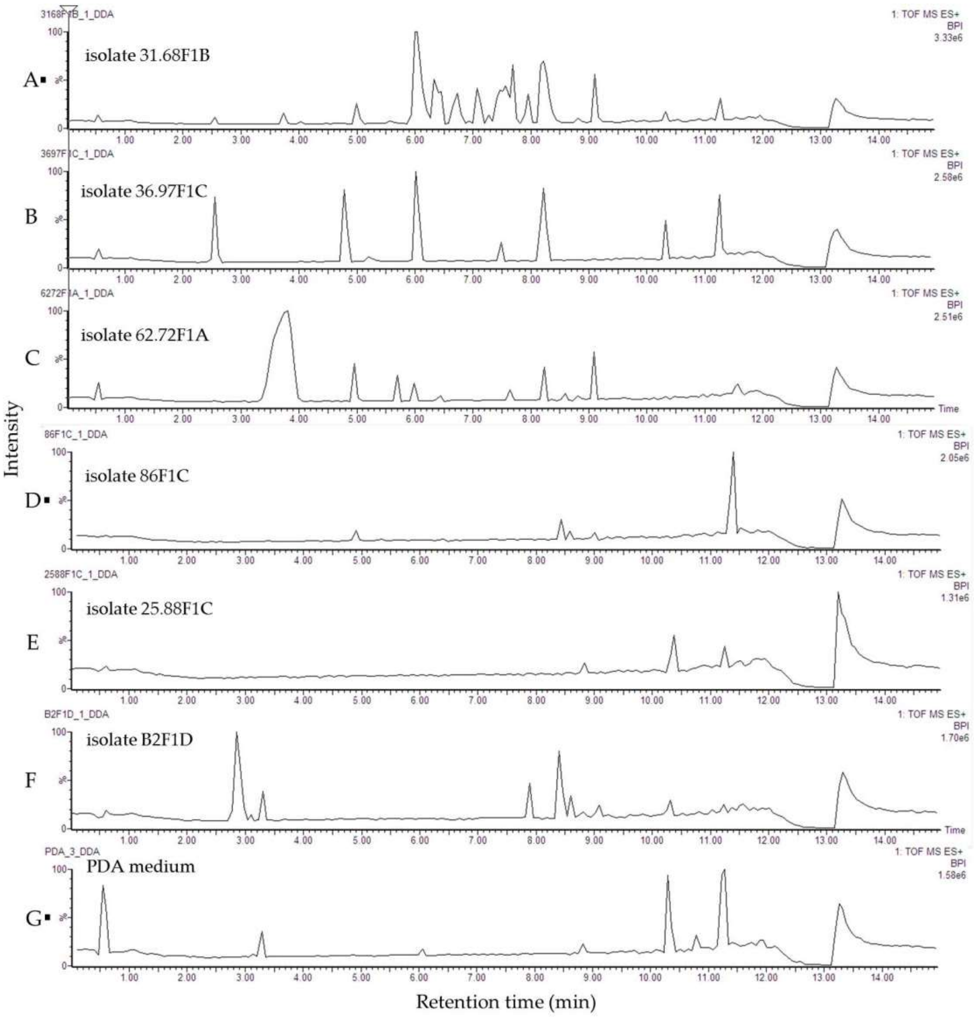

Figure S4. Base peak chromatograms (acquired by an UPLC-QToF-MS/MS system in positive mode) of extract of (A) *Penicillium* sp. 31.68F1B, (B) *Penicillium* sp. 36.97F1C, (C) *Penicillium* sp. 62.72F1A, (D) *Cladosporium* sp. 86F1C, (E) *Emericellopsis* sp. 25.88F1C, (F) *Plectosphaerella* sp. B2F1D, and (G) PDA medium

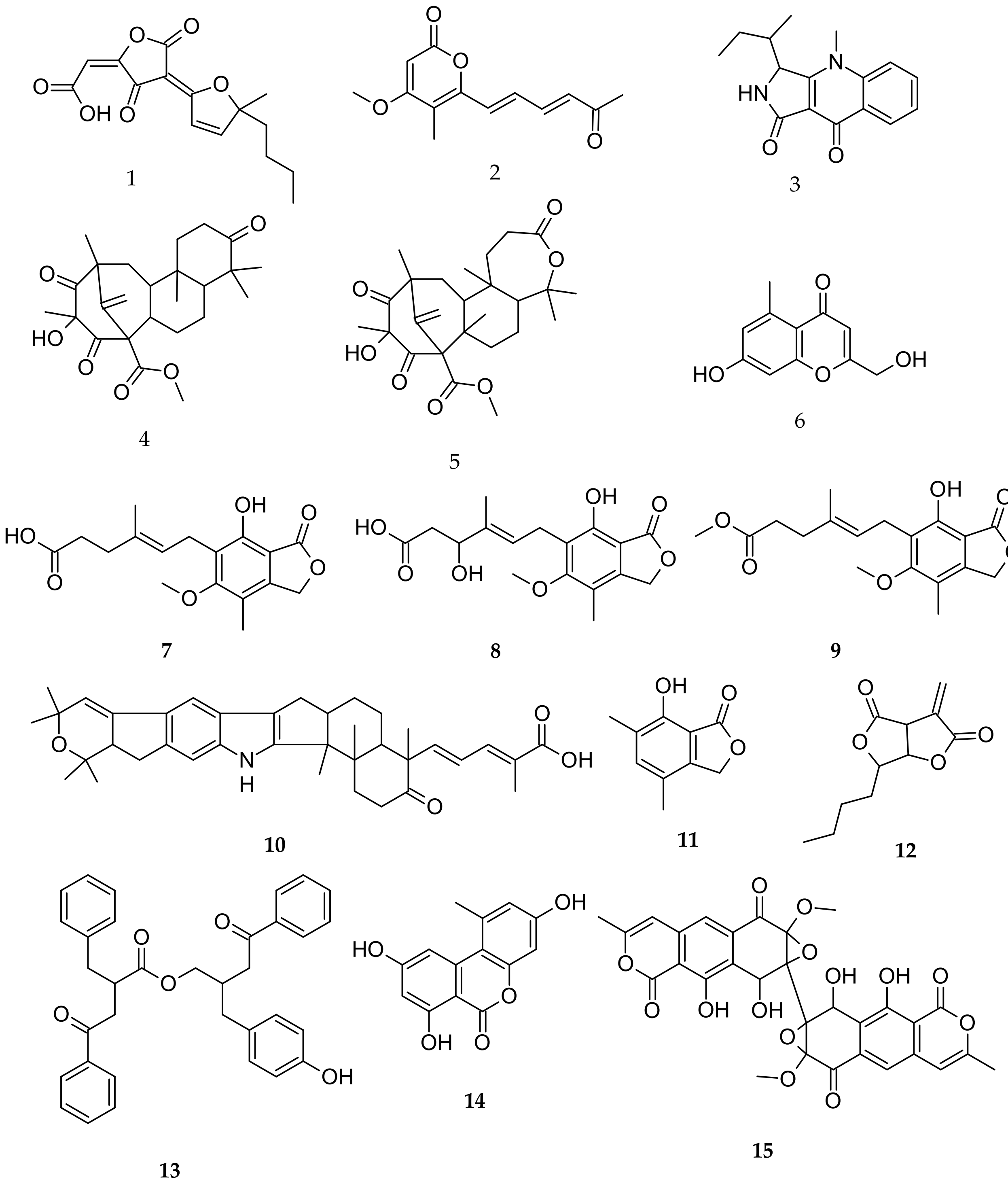

Figure S5. Chemical structures of metabolites annotated in the selected sea foam –derived fungi. Numbers correspond to putatively annotated known compounds, which are reported in Table S2.

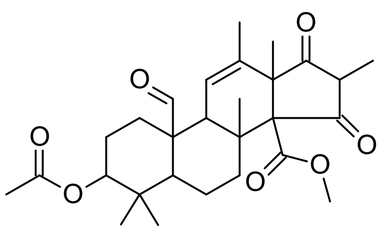

16

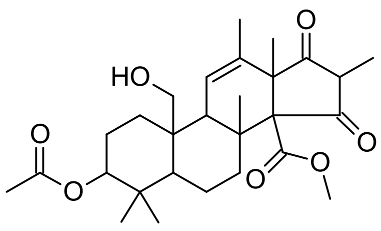

17

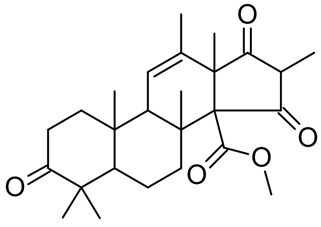

18

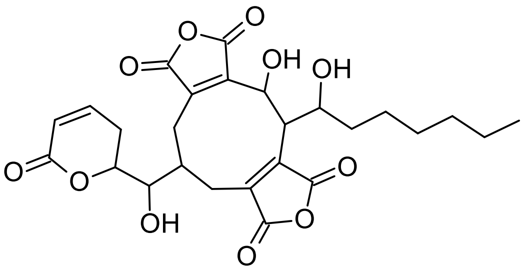

19

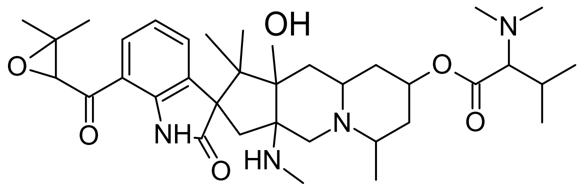

20

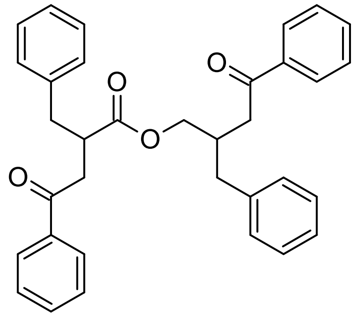

21

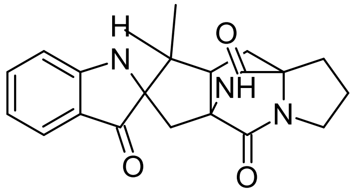

22

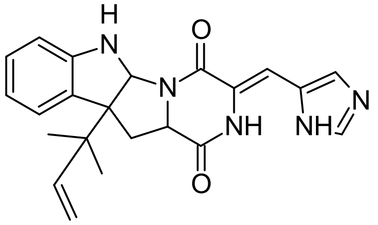

23

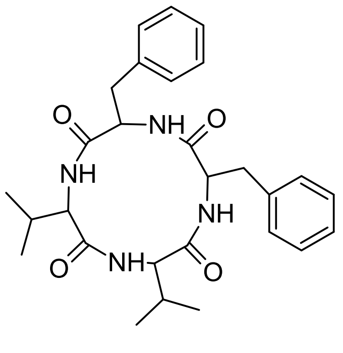

24

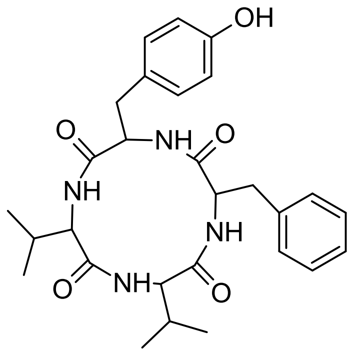

25

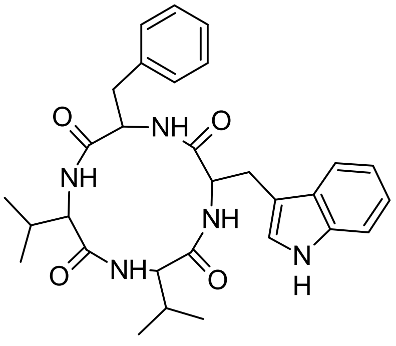

26

Figure S5. (continued)

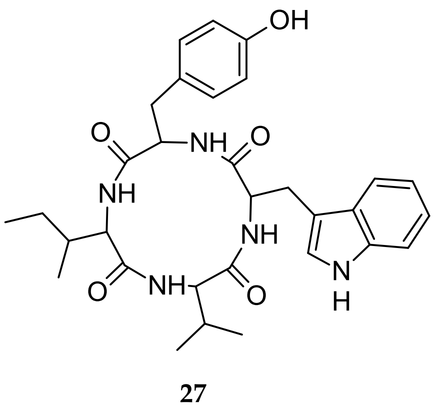

27

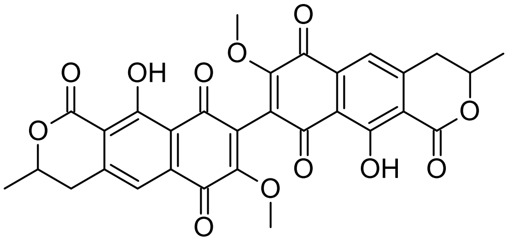

28

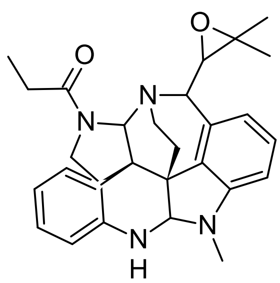

29

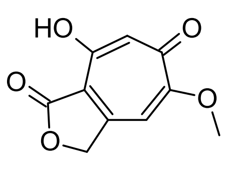

30

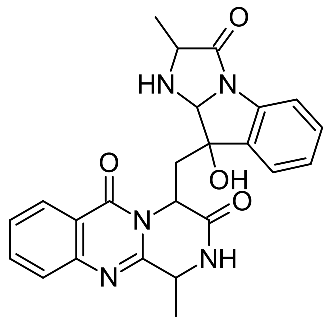

31

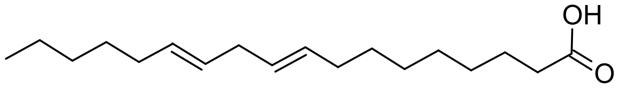

33

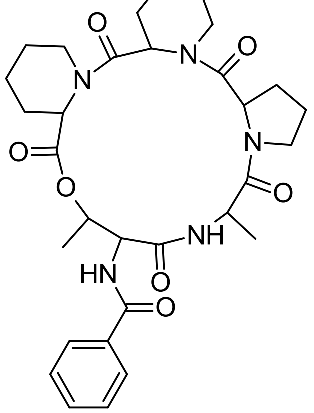

32

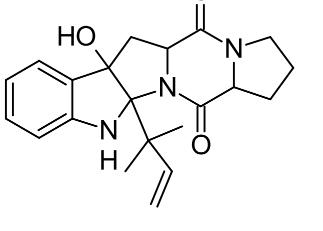

34

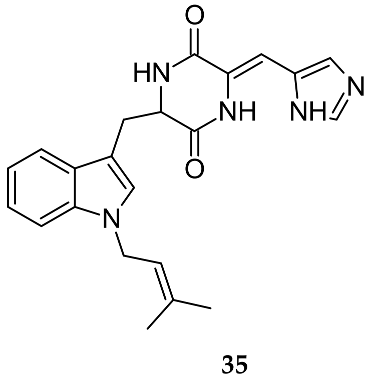

35

Figure S5. (continued)

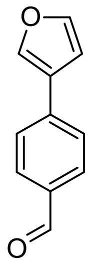

36

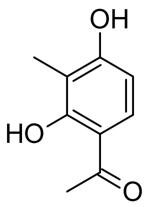

37

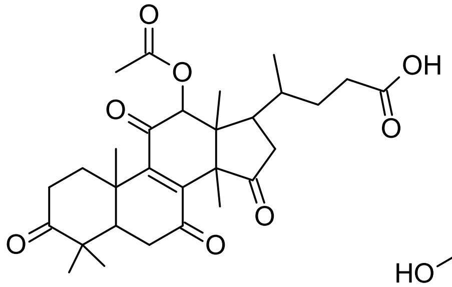

38

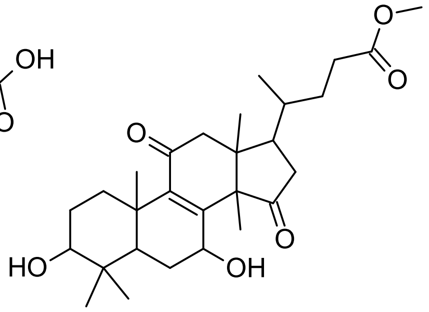

39

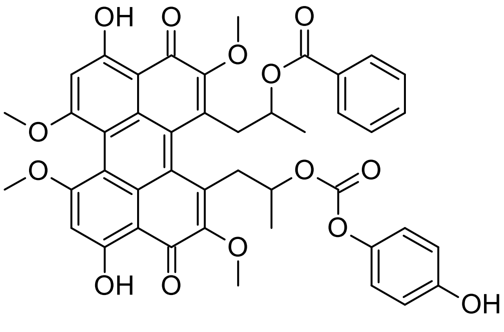

40

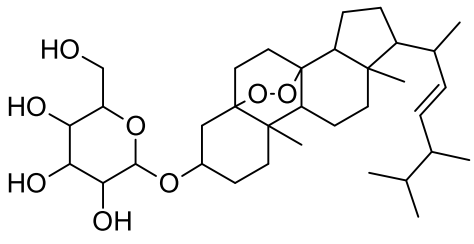

41

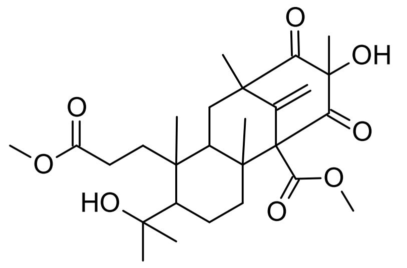

42

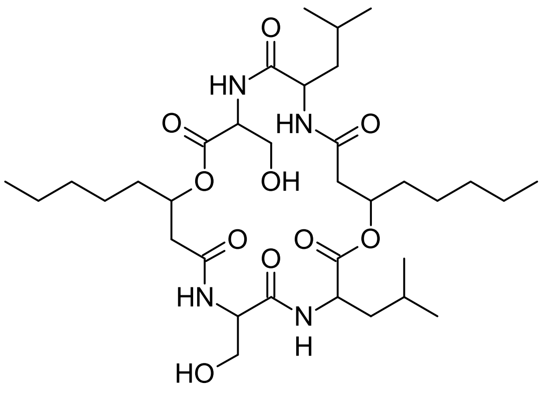

43

Figure S5. (continued)

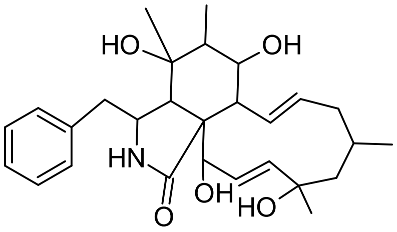

44

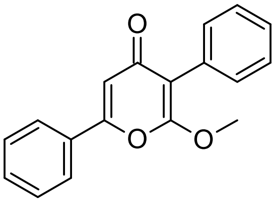

45

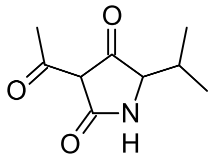

46

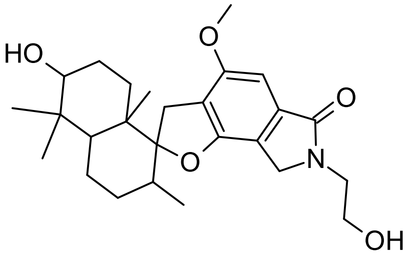

47

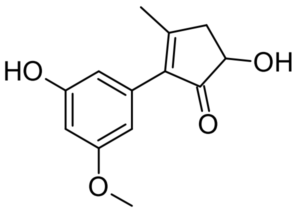

48

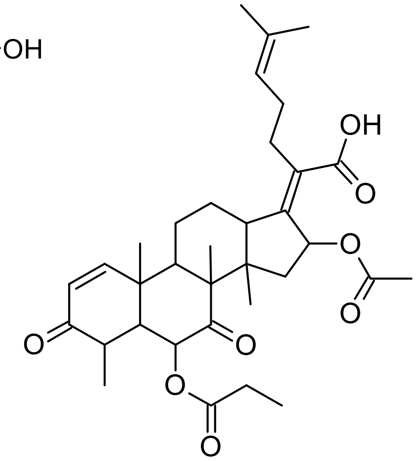

49

Figure S5. (continued)

References

1. Sumner, L.W.; Amberg, A.; Barrett, D.; Beale, M.H.; Beger, R.; Daykin, C.A.; Fan, T.W.-M.; Fiehn, O.; Goodacre, R.; Griffin, J.L., *et al.* Proposed minimum reporting standards for chemical analysis. *Metabolomics* **2007**, *3*, 211-221.
2. Arai, K.; Miyajima, H.; Mushiroda, T.; Yamamoto, Y. Metabolites of *Penicillium italicum* WEHMER : isolation and structures of new metabolites including naturally occurring 4-ylidene-acyltetronic acids, italicinic acid and italicic acid. *Chem. Pharm. Bull.* **1989**, *37*, 3229-3235.
3. Seiji, K.; Shin-ichi, K.; Shosuke, Y. Citreopyrones, new metabolites of two hybrid strains, KO 0092 and KO 0141, derived from the *Penicillium* species. *Chem. Lett.* **1997**, *26*, 33-34.
4. Del Valle, P.; Martínez, A.-L.; Figueroa, M.; Raja, H.A.; Mata, R. Alkaloids from the fungus *Penicillium spathulatum* as α-glucosidase Inhibitors. *Planta Med.* **2016**, *82*, 1286-1294.
5. Nong, X.-H.; Zhang, X.-Y.; Xu, X.-Y.; Sun, Y.-L.; Qi, S.-H. Alkaloids from *Xylariaceae* sp., a marine-derived fungus. *Nat. Prod. Commun.* **2014**, *9*, 1-2.
6. Maganhi, S.H.; Fill, T.P.; Rodrigues-Fo, E.; Caracelli, I.; Zukerman-Schpector, J. Preaustinoid A: a meroterpene produced by *Penicillium* sp. *Acta Cryst. E* **2009**, *65*, 1-10.
7. dos Santos, R.M.G.; Rodrigues-Fo, E. Further meroterpenes produced by *Penicillium* sp., an endophyte obtained from *Melia azedarach*. *Z. Naturforsch. C* **2003**, *58*, 663-669.
8. Kimura, Y.; Shiojima, K.; Nakajima, H.; Hamasaki, T. Structure and biological activity of plant growth regulators produced by *Penicillium* sp. No. 31f. *Biosci. Biotechnol. Biochem.* **1992**, *56*, 1138-1139.
9. Chen, Z.; Zheng, Z.; Huang, H.; Song, Y.; Zhang, X.; Ma, J.; Wang, B.; Zhang, C.; Ju, J. Penicacids A–C, three new mycophenolic acid derivatives and immunosuppressive activities from the marine-derived fungus *Penicillium* sp. SOF07. *Bioorg. Med. Chem. Lett.* **2012**, *22*, 3332-3335.
10. Xu, X.; Zhang, X.; Nong, X.; Wang, J.; Qi, S. Brevianamides and mycophenolic acid derivatives from the deep-Sea-derived fungus *Penicillium brevicompactum* DFFSCS025. *Mar. Drugs* **2017**, *15*, 1-10.
11. Singh, S.B.; Ondeyka, J.G.; Jayasuriya, H.; Zink, D.L.; Ha, S.N.; Dahl-Roshak, A.; Greene, J.; Kim, J.A.; Smith, M.M.; Shoop, W., *et al.* Nodulisporic acids D−F: structure, biological activities, and biogenetic relationships. *J. Nat. Prod.* **2004**, *67*, 1496-1506.
12. Miao, C.; Hu, J.; Zhai, Y.; Song, F.; Xuan, Q.; Chen, Y.; Wu, S. Identification and secondary metabolites of endophytic fungus PR20 from *Paeonia delavayi*. *Nat. Prod. Res. Dev.* **2012**, *24*, 1339-1342.
13. Zhang, W.; Xu, L.; Yang, L.; Huang, Y.; Li, S.; Shen, Y. Phomopsidone A, a novel depsidone metabolite from the mangrove endophytic fungus *Phomopsis* sp. A123. *Fitoterapia* **2014**, *96*, 146-151.
14. Sakata, K.; Masago, H.; Sakurai, A.; Takahashi, N. Isolation of aspirochlorine (=antibiotic A30641) possessing a novel dithiodiketopiperazine structure from *Aspergillus flavus*. *Tetrahedron Lett.* **1982**, *23*, 2095-2098.
15. McCorkindale, N.J.; Wright, J.L.C.; Brian, P.W.; Clarke, S.M.; Hutchinson, S.A. Canadensolide - an antifungal metabolite of *Penicillium canadense*. *Tetrahedron Lett.* **1968**, *9*, 727-730.
16. Liu, Z.-G.; Bao, L.; Liu, H.-W.; Ren, J.-W.; Wang, W.-Z.; Wang, L.; Li, W.; Yin, W.-B. Chemical diversity from the Tibetan Plateau fungi *Penicillium kongii* and *P. brasilianum*. *Mycology* **2018**, *9*, 10-19.
17. Guo, L.; Niu, S.; Chen, S.; Liu, L. Diaporone A, a new antibacterial secondary metabolite from the plant endophytic fungus *Diaporthe* sp. *J. Antibiot.* **2020**, *73*, 116-119.
18. Igarashi, Y.; Kuwamori, Y.; Takagi, K.; Ando, T.; Fudou, R.; Furumai, T.; Oki, T. Xanthoepocin, a new antibiotic from *Penicillium simplicissimum* IFO5762. *J. Antibiot.* **2000**, *53*, 928-933.
19. Nielsen, K.F.; Dalsgaard, P.W.; Smedsgaard, J.; Larsen, T.O. Andrastins A−D, *Penicillium roqueforti* metabolites consistently produced in blue-mold-ripened cheese. *J. Agric. Food Chem.* **2005**, *53*, 2908-2913.
20. Chen, R.-D.; Yan, Z.; Zou, J.-H.; Wang, N.; Dai, J.-G. Rubratoxin C, a new nonadride derivative from an endophytic fungus *Penicillium* sp. F-14. *Chin. Chem. Lett.* **2014**, *25*, 1308-1310.
21. Tsuda, M.; Kasai, Y.; Komatsu, K.; Sone, T.; Tanaka, M.; Mikami, Y.; Kobayashi, J.i. Citrinadin A, a novel pentacyclic alkaloid from marine-derived fungus *Penicillium citrinum*. *Org. Lett.* **2004**, *6*, 3087-3089.
22. Birch, A.J.; Wright, J.J. Studies in relation to biosynthesis—XLII: the structural elucidation and some aspects of the biosynthesis of the brevianamides-A and -E. *Tetrahedron* **1970**, *26*, 2329-2344.
23. Du, L.; Li, D.; Zhu, T.; Cai, S.; Wang, F.; Xiao, X.; Gu, Q. New alkaloids and diterpenes from a deep ocean sediment derived fungus *Penicillium* sp. *Tetrahedron* **2009**, *65*, 1033-1039.

24. Ali, H.; Ries, M.I.; Lankhorst, P.P.; van der Hoeven, R.A.M.; Schouten, O.L.; Noga, M.; Hankemeier, T.; van Peij, N.N.M.E.; Bovenberg, R.A.L.; Vreeken, R.J., *et al.* A non-canonical NRPS is involved in the synthesis of fungisporin and related hydrophobic cyclic tetrapeptides in *Penicillium chrysogenum*. *PLOS ONE* **2014**, *9*, 1-10.
25. Day, W.; Just, G.; Blank, F. Metabolites of pathogenic fungi. II: the Isolation of xanthomegnin from *Trichophyton Megnini*. *J. Invest. Dermatol.* **1963**, *40*, 133-137.
26. Dalsgaard, P.W.; Blunt, J.W.; Munro, M.H.; Frisvad, J.C.; Christophersen, C. Communesins G and H, new alkaloids from the psychrotolerant fungus *Penicillium rivulum*. *J. Nat. Prod.* **2005**, *68*, 258-261.
27. Buckingham, J. Dictionary of Natural Products (on-line web edition). Available online: http://www.chemnetbase.com (accessed on 20 January 2020).
28. Zhang, H.; Liu, R.; Yang, J.; Li, H.; Zhou, F. Bioactive alkaloids of *Aspergillus fumigatus*, an endophytic fungus from *Astragalus membranaceus*. *Chem. Nat. Compd.* **2017**, *53*, 802-805.
29. Kawahara, T.; Takagi, M.; Shin-ya, K. Three new depsipeptides, JBIR-113, JBIR-114 and JBIR-115, isolated from a marine sponge-derived *Penicillium* sp. fS36. *J. Antibiot.* **2011**, *65*, 147-150.
30. Shan, W.-G.; Ying, Y.-M.; Yu, H.-N.; Liu, W.-H.; Zhan, Z.-J. Diketopiperazine alkaloids from *Penicillium* spp. HS-3, an endophytic fungus in *Huperzia serrata*. *Helv. Chim. Acta* **2010**, *93*, 772-776.
31. Kokubun, T.; Rozwadowski, Z.; Duddeck, H. Benzaldehyde derivatives from *Sarcodontia crocea*. *J. Nat. Prod.* **2007**, *70*, 1539-1541.
32. Prabpai, S.; Wiyakrutta, S.; Sriubolmas, N.; Kongsaeree, P. Antimycobacterial dihydronaphthalenone from the endophytic fungus *Nodulisporium* sp. of *Antidesma ghaesembilla*. *Phytochem. Lett.* **2015**, *13*, 375-378.
33. Kikuchi, T.; Matsuda, S.; Kadota, S.; Murai, Y.; Ogita, Z. Ganoderic acid D, E, F, and H and lucidenic acid D, E, and F, new triterpenoids from *Ganoderma lucidum*. *Chem. Pharm. Bull.* **1985**, *33*, 2624-2627.
34. Arnone, A.; Assante, G.; Merlini, L.; Nasini, G. Structure and stereochemistry of cladochrome D and E, novel perylenequinone pigments from *Cladosporium cladosporioides*. *Gazz. Chim. Ital.* **1989**, *119*, 557-559.
35. Bok, J.W.; Lermer, L.; Chilton, J.; Klingeman, H.G.; Towers, G.H.N. Antitumor sterols from the mycelia of *Cordyceps sinensis*. *Phytochemistry* **1999**, *51*, 891-898.
36. Duan, R.; Zhou, H.; Yang, Y.; Li, H.; Dong, J.; Li, X.; Chen, G.; Zhao, L.; Ding, Z. Antimicrobial meroterpenoids from the endophytic fungus *Penicillium* sp. T2-8 associated with *Gastrodia elata*. *Phytochem. Lett.* **2016**, *18*, 197-201.
37. Izawa, Y.; Hirose, T.; Shimizu, T.; Koyama, K.; Natori, S. Six new 10-pheynl-[11]cytochalasans, cytochalasins N - S from *Phomopsis* sp. *Tetrahedron* **1989**, *45*, 2323-2335.
38. Liu, K.; Wang, J.-L.; Wu, H.-B.; Wang, Q.; Bi, K.-L.; Song, Y.-F. A new pyranone from *Lenzites betulina*. *Chem. Nat. Compd.* **2012**, *48*, 780-781.
39. Lebrun, M.H.; Dutfoy, F.; Gaudemer, F.; Kunesch, G.; Gaudemer, A. Detection and quantification of the fungal phytotoxin tenuazonic acid produced by *Pyricularia oryzae*. *Phytochemistry* **1990**, *29*, 3777-3783.
40. Kamalov, L.S.; Aripova, S.F.; Isaev, M.I. Low-molecular-mass metabolites of fungi IV. the structures of stachybotrin A and stachybotral. *Chem. Nat. Compd.* **1999**, *35*, 82-85.
41. Chen, Y.; Chen, R.; Xu, J.; Tian, Y.; Xu, J.; Liu, Y. Two new altenusin/thiazole hybrids and a new benzothiazole derivative from the marine sponge-derived fungus *Alternaria* sp. SCSIOS02F49. *Molecules* **2018**, *23*, 1-8.
42. Pang, X.; Lin, X.; Wang, P.; Zhou, X.; Yang, B.; Wang, J.; Liu, Y. Perylenequione derivatives with anticancer activities isolated from the marine sponge-derived fungus, *Alternaria* sp. SCSIO41014. *Mar. Drugs* **2018**, *16*, 1-13.
43. Kong, F.-D.; Huang, X.-L.; Ma, Q.-Y.; Xie, Q.-Y.; Wang, P.; Chen, P.-W.; Zhou, L.-M.; Yuan, J.-Z.; Dai, H.-F.; Luo, D.-Q., *et al.* Helvolic acid derivatives with antibacterial activities against *Streptococcus agalactiae* from the marine-derived fungus *Aspergillus fumigatus* HNMF0047. *J. Nat. Prod.* **2018**, *81*, 1869-1876.
